# Melanocortin 1 Receptor Regulates Pathological and Physiological Cardiac Remodeling

**DOI:** 10.1161/JAHA.124.037961

**Published:** 2025-02-08

**Authors:** Anni Suominen, Aino Suni, Saku Ruohonen, Zoltán Szabó, Lotta Pohjolainen, Minying Cai, Eriika Savontaus, Virpi Talman, Risto Kerkelä, Petteri Rinne

**Affiliations:** ^1^ Research Centre for Integrative Physiology & Pharmacology, Institute of Biomedicine University of Turku Finland; ^2^ Drug Research Doctoral Programme (DRDP) University of Turku Finland; ^3^ Research Unit of Biomedicine and Internal Medicine, Department of Pharmacology and Toxicology University of Oulu Finland; ^4^ Drug Research Program and Division of Pharmacology and Pharmacotherapy, Faculty of Pharmacy University of Helsinki Finland; ^5^ Department of Chemistry and Biochemistry University of Arizona Tucson AZ USA; ^6^ Turku Center for Disease Modeling University of Turku Finland; ^7^ Unit of Clinical Pharmacology Turku University Hospital Turku Finland; ^8^ Medical Research Center Oulu Oulu University Hospital and University of Oulu Finland

**Keywords:** cardiac hypertrophy, cardiomyocyte, heart failure, melanocortin 1 receptor, Basic Science Research, Myocardial Biology, Hypertrophy, Heart Failure

## Abstract

**Background:**

The melanocortin 1 receptor (MC1R) is abundantly expressed in the skin and leukocytes, where it regulates skin pigmentation and inflammatory responses. Recently, MC1R was also found in the heart, but its functional role has remained unknown. We aimed to investigate whether MC1R is involved in the regulation of pathological or physiological cardiac remodeling.

**Methods and Results:**

Recessive yellow mice, as a model of global MC1R deficiency, and cardiomyocyte‐specific MC1R knockout mice were subjected to transverse aortic constriction or voluntary wheel running to induce pathological or physiological cardiac hypertrophy, respectively. Mice were phenotyped for cardiac structure and function by echocardiography, histology, and quantitative PCR analysis. H9c2 cells and neonatal mouse ventricular cardiac myocytes were used as in vitro models to investigate the effects of pharmacological MC1R activation on hypertrophy‐related responses. We found that the expression of MC1R progressively declines in the failing mouse heart. MC1R recessive yellow mice showed blunted hypertrophic response to transverse aortic constriction–induced pressure overload and exercise training. This phenotype was recapitulated in MC1R knockout mice, demonstrating that MC1R deficiency specifically in cardiomyocytes is responsible for the antihypertrophic effect. However, MC1R knockout mice subjected to pressure overload showed left ventricular dilatation that was associated with reduced ejection fraction and changes in left ventricular diastolic function. At the molecular level, the mRNA expression of myosin heavy chain β was upregulated in the hearts of MC1R knockout mice. In contrast, selective activation of MC1R promoted hypertrophic responses in cultured cardiomyocytes.

**Conclusions:**

Cardiomyocyte‐specific MC1R deficiency attenuates physiological and pathological cardiac hypertrophy in mice, while pharmacological activation of MC1R promotes cardiomyocyte hypertrophy.

## Graphical abstract

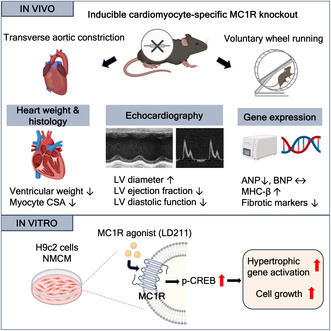

**Nonstandard Abbreviations and Acronyms**
CREB: cAMP response element–binding protein
CSA: cross‐sectional area
hiPSC‐CM: human‐induced pluripotent stem cell‐derived cardiomyocytes
LVEDD: left ventricular end‐diastolic dimension
MC1R: melanocortin 1 receptor
Mc1r‐cKO: cardiomyocyte‐specific melanocortin 1 receptor knockout
Mc1r^e/e^
: melanocortin 1 receptor recessive yellow
MHC: myosin heavy chain
Myh6‐MCM: Myh6‐MerCreMer transgenic
NMCM: neonatal mouse ventricular cardiac myocyte
TAC: transverse aortic constriction
WT: wild‐type

**Research Perspective**

**What Is New?**

- Melanocortin 1 receptor (MC1R), which is a well‐known genetic determinant of skin and hair color, was found to be also expressed in the mouse heart and downregulated during pathological cardiac remodeling.
- Global and cardiomyocyte‐specific deficiency of MC1R attenuated cardiac hypertrophy after both pressure overload and exercise training.
- Loss of MC1R in cardiomyocytes was, however, associated with left ventricular dilatation and compromised cardiac function following pressure overload.

**What Question Should Be Addressed Next?**

- From a translational perspective, several loss‐of‐function variants of MC1R have been identified in humans and analogues of naturally occurring α‐melanocyte‐stimulating hormone, which have agonistic activity at MC1R, and have been recently approved for clinical use in different therapeutic areas.
- Even if targeting of MC1R would not translate into therapeutic benefits in the management of human heart failure, our findings highlight the importance of investigating the possible cardiac effects of genetic MC1R deficiency and MC1R‐activating therapies.

Melanocortin receptors are a family of 5 G‐protein coupled receptors named from MC1R to MC5R. These receptors are widely expressed in different tissues, and they regulate many different physiological functions by interacting with their cognate ligands known as melanocortin peptides, which include melanocyte‐stimulating hormones (α‐, β‐ and γ‐melanocyte‐stimulating hormone) and adrenocorticotropic hormone.^1^, ^2^, ^3^ Among different melanocortin receptor subtypes, MC1R was the first receptor member to be cloned, and it is abundantly expressed in the skin, where it regulates melanin formation and skin pigmentation.^4^, ^5^ The *MC1R* gene is highly polymorphic in humans, and several loss‐of‐function variants have been identified and linked to the red hair color phenotype.^5^, ^6^ Likewise, in mice, naturally occurring recessive alleles have been identified in the *MC1R* gene and found to associate with yellow coat color.^7^ For example, the recessive yellow (Mc1r^e/e^) mice that carry a nonfunctional MC1R due to a single base deletion mutation is used as an experimental tool to model global MC1R deficiency.^6^ It has been shown that the phenotype of these mice extends beyond a defect in pigmentation, as they have, for example, changes in their vasculature including endothelial dysfunction and arterial stiffness.^8^ This phenotype is also recapitulated in humans carrying loss‐of‐function variants of *MC1R*.^8^ In addition, MC1R regulates inflammatory responses through its wide expression in the immune system cells like monocytes and macrophages.^9^ Consequently, in atherosclerosis, which is a chronic inflammatory disease of arteries, global deficiency of MC1R signaling in Mc1r^e/e^ mice promoted accumulation of leukocytes in the arteries and systemic inflammation and accelerated the development of atherosclerotic plaques.^10^ Intriguingly, previous studies have reported MC1R expression in developing human and mouse hearts,^11^ as well as in adult rat hearts.^12^, ^13^ However, the functional role of MC1R in the heart has remained elusive.

Following the previously discovered expression of MC1R in the heart, we first found that the expression of MC1R in the mouse heart declines during the development of pathological cardiac hypertrophy. Based on this initial finding, we hypothesized that MC1R is involved in the regulation of cardiac remodeling. To test this hypothesis, we used Mc1r^e/e^ mice, as a model of global MC1R deficiency and subjected them to models of pathological and physiological cardiac hypertrophy. Mc1r^e/e^ mice displayed features of attenuated cardiac hypertrophy, which led us to engineer a cardiomyocyte‐specific MC1R knockout mouse model to investigate whether the observed phenotype was dependent on dysfunctional MC1R signaling in cardiomyocytes. Indeed, cardiomyocyte‐specific deletion of MC1R caused a similar phenotype with attenuated cardiac hypertrophy in response to pathological stimuli and endurance training. In line with these findings, pharmacological activation of MC1R promoted cardiomyocyte hypertrophy in vitro. Taken together, these experimental data uncover a functional role for MC1R in the heart and demonstrate that its integrity is essential for pathological and physiological cardiac remodeling.

## Methods

The authors declare that all supporting data are available within the main body of the manuscript and in the online supplementary data.

### Mice and Study Design

Our study exclusively examined male mice, which are known to be more susceptible for developing cardiac hypertrophy and heart failure.^14^, ^15^ It is unknown whether the findings of the present study are relevant for female mice.

All experiments were performed on group‐housed adult male mice unless otherwise indicated. Mice were maintained on a 12‐hour light/dark cycle with free access to food (Teklad Global diet, Envigo, No. 2916C) and tap water. The experiments were approved by the national Animal Experiment Board in Finland (License Nos. ESAVI‐438/04.10.03/2012, ESAVI/6280/04.10.07/2016, ESAVI/1260/2020, and ESAVI/45421/2022) and conducted in accordance with Directive 2010/63/EU of the European Parliament on the protection of animals used for scientific purposes and with the institutional and national guidelines for the care and use of laboratory animals. Sample sizes were empirically determined on the basis of previous experience with the experimental models. Where possible, experiments were conducted and analyzed by blinded researchers.

Mc1r^e/e^ mice, which lack functional MC1R due to a single base deletion mutation in the *MC1R* gene, were obtained from the Jackson Laboratory (Bar Harbor, ME; Strain No. 000060) and used as a model of global MC1R deficiency. All experiments were performed on adult (4–6 months) Mc1r^e/e^ mice. Age‐matched nonmutant littermates (wild‐type [WT]) were uses as controls.

To generate inducible cardiomyocyte‐specific MC1R knockout (Mc1r‐cKO) mice, *MC1R* floxed mice (Jackson Laboratory; Strain No. 029239)^16^ were intercrossed with tamoxifen‐inducible Myh6‐MerCreMer transgenic (Myh6‐MCM; Jackson Laboratory; Strain No. 005657) mice.^17^ All mice were on C57Bl/6J background. The generation of *MC1R* floxed mice has been previously described, and these mice have been successfully intercrossed with different Cre transgenic mouse lines.^13^, ^16^ Six‐week‐old (range, 5–7 weeks) male mice were treated with tamoxifen (20 mg/kg IP; Cayman Chemicals, Ann Arbor, MI; No. 13258) on 4 consecutive days to induce Cre‐mediated recombination in cardiomyocytes, as previously described.^17^, ^18^ Age‐matched Cre‐positive Mc1r^wt/wt^ (referred to as Myh6‐MCM) mice were used as controls and treated with tamoxifen, as described above. Mice were allowed to recover at least for 7 days from tamoxifen treatment before any experimentation. At the end of the experiment, genomic DNA was extracted from heart samples and genotyped using the following primers: ACC ACT GCG TGC TAT CCT G (5′ forward), ACC CCT TCC CTT GAG GAG T (5′ reverse) and GAA CTC TGA GGT CAC TAT TTT CTG GAG A (3′ reverse). The positions of the primers in the *Mc1r* gene are presented in Figure S1. Genomic DNA samples from the liver, spleen, and skeletal muscle were also genotyped to verify that the desired recombination occurs specifically in the heart (Figure S1).

### Models of Pathological and Physiological Cardiac Hypertrophy

To induce pathological cardiac hypertrophy, Mc1r^e/e^ (n=13) and Mc1r‐cKO (n=7) mice and their controls (n=7 for WT and Myh6‐MCM) were subjected to transverse aortic constriction (TAC), as previously described.^18^ Briefly, mice were anesthetized with ketamine (110 mg/kg IP) and xylazine (15 mg/kg IP), intubated (Venflon Pro cannula; BD; No. 393202) and ventilated (Minivent type 845; Harvard Apparatus) during the surgery. A median sternotomy was performed and the thymus was gently repositioned, and peri‐aortic fat tissue was carefully removed to expose the aortic arch and its primary branches. A curved 27‐g needle was used as a spacer for ligation of the aortic arch between the brachiocephalic trunk and left common carotid artery with a 7‐0 silk suture. The thoracic cage and skin were thereafter closed with 6‐0 surgical silk sutures. Sham‐operated mice (n=4–7/genotype) underwent exactly the same procedure without constriction of the aorta. Buprenorphine (0.05 mg/kg SC, 2×/d for 3 days) and carprofen (5 mg/kg SC, 1×/d for 3 days) were given for peri‐ and postoperative analgesia. Mortality rates during and after the operation are reported in Table S1.

As another model of pathological cardiac hypertrophy, Mc1r‐cKO (n=8) and Myh6‐MCM (n=7) mice were subjected to subcutaneous infusion of angiotensin II (1.4 mg/kg per d) for 4 weeks using osmotic minipumps (Alzet, model 1004).^18^, ^19^ Mice were anesthetized with isoflurane (4% for induction and 2% for maintenance) and a dorsal, midline incision was made to create a subcutaneous pocket for the insertion of the osmotic minipump. Thereafter, the skin was closed with sutures, and buprenorphine (0.05 mg/kg SC, 2×/d for 3 days) and carprofen (5 mg/kg SC, 1×/d for 3 days) were given for peri‐ and postoperative analgesia. Sham‐operated mice (n=6–7 mice/genotype) underwent the same procedure without implantation of minipump.

To induce physiological cardiac hypertrophy, Mc1r^e/e^ (n=11) and Mc1r‐cKO (n=11) mice and their controls (n=13 for WT and n=8 for Myh6‐MCM) were subjected to voluntary wheel running for 5 weeks. For this purpose, mice were individually housed and a running wheel with magnetic counter (Low Profile Wireless Running Wheel for Mice, ENV‐004; Med Associates Inc.) was placed into each home cage. Individual running data were collected and stored in wheel manager software (SOF‐860; Med Associates Inc.). A nonexercising control group (referred to as sedentary mice; n=4–5 mice/genotype) underwent the same procedure but their running wheels were made nonrotating using special stoppers.

At the end of the experiments, mice were euthanized via CO_2_ asphyxiation, the heart was excised and rinsed in PBS, and ventricular weight was measured after removing the atria.

### Echocardiography

Cardiac structure and function were assessed by transthoracic echocardiography (Vevo 2100; Visual Sonics Inc., Toronto, Ontario, Canada) before the start of the hypertrophy induction and at the end of the experiment under isoflurane anesthesia (4% for induction and 2% for maintenance). Parasternal short‐ and long‐axis images (B‐ and M‐mode) as well as transmitral pulsed‐wave and tissue Doppler images were recorded and analyzed with Vevo software (Vevo LAB 5.5.0) by a blinded observer. Pulsed‐wave Doppler images were acquired to measure the velocities of E and A waves of transmitral flow as well as isovolumetric relaxation time and E wave deceleration time using the apical 4‐chamber for targeting and positioning the Doppler window in the center of the main jet. Tissue Doppler images were recorded to measure mitral annular tissue movement during early (e′) and late (a′) left ventricular (LV) filling and the combined ratio of E/e′. Parasternal short‐ and long‐axis B‐mode images were used to calculate global longitudinal, circumferential, and radial strain and strain rate by speckle‐tracking–based analysis (VevoStrain software). Relative wall thickness was calculated as follows: (LV posterior wall thickness+LV anterior wall thickness)/LV end‐diastolic diameter.

### Histology, Immunohistochemistry, and Immunofluorescence Staining

Mouse heart samples were transversely cut in the midline of the base‐apex axis, and the superior part of the heart was fixed in 10% formalin overnight, embedded in paraffin, and cut into 5‐μm‐thick serial sections. Sections were stained with hematoxylin and eosin and Picro‐Sirius red (Abcam, No. ab245887) to measure the cross‐sectional area (CSA) of cardiomyocytes, cardiomyocyte length, and cardiac fibrosis level in the LV free wall at the level of the papillary muscles. For CSA quantification, at least 100 individual cells with well‐defined cell membranes and visible cell nuclei were selected and measured in fields of longitudinally oriented cardiomyocytes. For MC1R immunohistochemistry, heart sections were deparaffinized, rehydrated, and subjected for antigen retrieval, as previously described.^13^, ^20^ After quenching (1% H_2_O_2_, 10 minutes) and blocking (5% normal horse serum, 60 minutes), sections were incubated overnight with a primary antibody against MC1R (Elabscience, Texas; No. E‐AB‐15765) followed by detection with ImmPRESS HRP Horse Anti‐Rabbit IgG PLUS Polymer Kit (Vector Labs, Burlingame, CA; No. MP‐7801‐15). For isotype control, a consecutive heart section was treated similarly except that the primary MC1R antibody was replaced by purified normal rabbit IgG (Novus Biologicals, Littleton, CO; No. NB810‐56910). For immunofluorescence, heart sections were stained with primary antibodies against MC1R and sarcomeric α‐actinin (Merck Life Science; No. A7811), α‐smooth muscle actin (α‐SMA, Merck Life Science; No. A5228) or CD31 (R&D Systems; No. AF3628) followed by detection with fluorochrome‐conjugated secondary antibodies (Alexa Fluor 488 and Alexa Fluor 647; Jackson ImmunoResearch, West Grove, PA). Sections were counterstained with hematoxylin (CarlRoth) or DAPI, cover‐slipped, and then scanned with Pannoramic 250 or Pannoramic Midi digital slide scanner (3DHISTECH Kft, Budapest, Hungary). Image analysis was performed using ImageJ software (National Institutes of Health, Bethesda, MD).

### Cell Culture and Treatments

Rat heart myoblast H9c2 (2‐1) cells (ATCC, CRL‐1446), neonatal mouse ventricular cardiac myocytes (NMCMs) and human‐induced pluripotent stem cell‐derived cardiomyocytes (hiPSC‐CMs) were cultured, as previously described.^21^, ^22^, ^23^ To investigate the effect of hypertrophic stimuli on *MC1R* expression, hiPSC‐CMs were differentiated from the iPS (IMR90)‐4 cell line (WiCell, Madison, WI) using small‐molecule induction, matured until day 30 after the initiation of the differentiation and treated with endothelin 1 for 24 hours or subjected to mechanical stretching for 24 or 48 hours, as previously described.^24^, ^25^ To study the effects of MC1R activation, H9c2 cells and NMCMs were treated with the selective MC1R agonist LD211 (compound 28 in the original publication).^26^ To study the effects of MC1R activation under hypertrophic conditions, H9c2 cells were treated with LD211 in the absence or presence of angiotensin II (Abcam; No. ab120183) for 24 hours. To inhibit the p‐38 and CREB (cAMP response element–binding protein) pathways, H9c2 cells were treated with TAK‐715 (5 μM; CaymanChemical; No. 26170) or 666‐15 (1 μM; MedChemExpress; No. HY‐101120), respectively, for 30 minutes before stimulation with LD211.

### cAMP Determination

To measure intracellular cAMP concentrations, H9c2 cells were harvested, pipetted into a 96‐well OptiPlate (6000 cells/well) and stimulated with different concentrations of LD211 for 30 minutes in the presence of 3‐isobutyl‐1‐methylxanthine (0.1 mM). cAMP levels were determined using a LANCE Ultra cAMP Detection Kit (PerkinElmer; No. TRF0262) according to the manufacturer's instructions. To detect possible Gαi‐evoked inhibition of intracellular cAMP production, cells were treated with different concentrations of LD211 in the presence of forskolin (1 μM).

### Ca^2+^ Mobilization Assay

To investigate the actions of LD211 on Ca^2+^ mobilization, H9c2 cells were seeded into a CellCarrier Ultra 96‐well plate (8000 cells/well), loaded with Fluo‐4 Direct calcium detection reagent (Thermo Fisher; No. F10471) and measured for drug‐evoked changes in fluorescence using Ensight Multimode Plate Reader (excitation wavelength 494 nm, emission wavelength 516 nm; PerkinElmer), as previously described.^23^ The muscarinic receptor agonist carbachol (100 μM) was used as a positive control.

### [^3^H]‐Leucine Incorporation Assay

To estimate the rate of protein synthesis, H9c2 cells were cultured in DMEM containing L‐[4,5‐^3^H]‐leucine (1 μCi/mL; PerkinElmer) and treated with LD211 (1 pM, 0.1 nM, 10 nM, or 1 μM) for 24 hours. In separate experiments, H9c2 cells were pretreated for 30 minutes with the p‐38 inhibitor TAK‐715 (5μM; CaymanChemical; No. 26170) or the CREB inhibitor 666‐15 (1μM; MedChemExpress; No. HY‐101120) before stimulation with LD211. After 24 hours, cells were rinsed with PBS and the protein were precipitated, lysed, and used for radioactivity measurement with automatic liquid scintillation counter (Hidex 600 SL; Hidex), as previously described.^23^

### RNA Isolation, cDNA Synthesis, and Quantitative Reverse Transcription Polymerase Chain Reaction

H9c2, NMCMs, and hiPSC‐CMs were collected into QIAzol Lysis Reagent or Trizol Reagent (Invitrogen), and total RNA was extracted using Direct‐zol RNA Miniprep or Microprep (Zymo Research, Irvine, CA). Heart samples were first homogenized in QIAzol Lysis Reagent using the Qiagen TissueLyser LT Bead Mill (QIAGEN, Venlo, Netherlands), and total RNA was thereafter extracted using Direct‐zol RNA Miniprep. RNA was reverse‐transcribed to cDNA (PrimeScript RT reagent kit; Takara Clontech) and quantitative real‐time polymerase chain reaction (qPCR) was performed with SYBR Green protocols (Kapa Biosystems, Wilmington, MA) and a real‐time PCR detection system (7300 Real‐Time PCR system; Applied Biosystems).^20^, ^27^ Target gene expression was normalized to a housekeeping gene (ribosomal protein S18, GAPDH, or β‐actin) using the comparative ΔCt method, and results are presented as relative transcript levels. Primer sequences are presented in Tables S2 through S4.

### Western Blot

Cell and tissue samples were lysed in RIPA buffer supplemented with protease and phosphatase inhibitors (Complete Mini, Roche; and Halt Phosphatase Inhibitor Cocktail, Thermo Fisher). Aliquots of total protein were separated by SDS‐PAGE and transferred to nitrocellulose or polyvinylidene difluoride membranes. After blocking with 5% nonfat milk or 3% BSA, membranes were incubated with primary antibodies overnight at 4 °C. The following primary antibodies were used: anti‐MC1R antibody (Alomone Labs, Jerusalem, Israel; No. AMR‐025), anti–phospho‐JNK (Cell Signaling Tech, Frankfurt, DE; No. 4668), anti– c‐Jun N‐terminal kinase (R&D Systems; No. AF‐1387), anti–phospho‐extracellular signal‐related kinases (ERK)1/2 (Cell Signaling Tech; No. 9107), anti–extracellular signal‐related kinases 1/2 (Cell Signaling Tech; No. 9106), anti–phospho‐p38 (Cell Signaling Tech; No. 9215), anti‐p38 (Cell Signaling Tech; No. 9212), anti–phospho‐CREB (Thermo Fisher; No. MA5‐11192) and anti‐CREB (Cell Signaling Tech; No. 9197). After primary incubation, membranes were washed and incubated with horseradish peroxidase–conjugated anti‐IgG secondary antibodies (Cell Signaling Tech) for 1 hour at room temperature followed by detection using a chemiluminescence system (Pierce ECL Western Blotting Substrate, Thermo Fisher) and Sapphire Biomolecular Imager (Azure Biosystems). The results for target protein expression were normalized to β‐actin (Merck Life Science; No. 2066) or vinculin (BioRad; No. MCA465GA) expression to correct for loading.

### Statistical Analysis

Statistical analyses were performed with Prism 9 and 10 (GraphPad Software, La Jolla, CA). Statistical significance between the experimental groups was determined by unpaired Student's *t* test and 1‐way ANOVA followed by Dunnett post hoc tests or 2‐way ANOVA followed by Šídák's post hoc tests. Data for running distance and changes in echocardiographic parameters in the running wheel experiments were analyzed using 2‐way repeated‐measures ANOVA. The normality of the data was evaluated using the D'Agostino and Pearson omnibus normality test. Data that did not pass D'Agostino and Pearson normality test or had fewer than 6 samples per group were analyzed using the Mann–Whitney *U* test or Kruskal–Wallis test followed by Dunn's post hoc tests. Possible outliers in the data sets were identified using the regression and outlier removal method at a Q‐level of 1%. Data are expressed as mean± SEM. Results were considered significant for *P*<0.05.

## Results

### MC1R Is Expressed in the Mouse Heart and Regulated by Hypertrophic Stimuli

**Figure 1:**
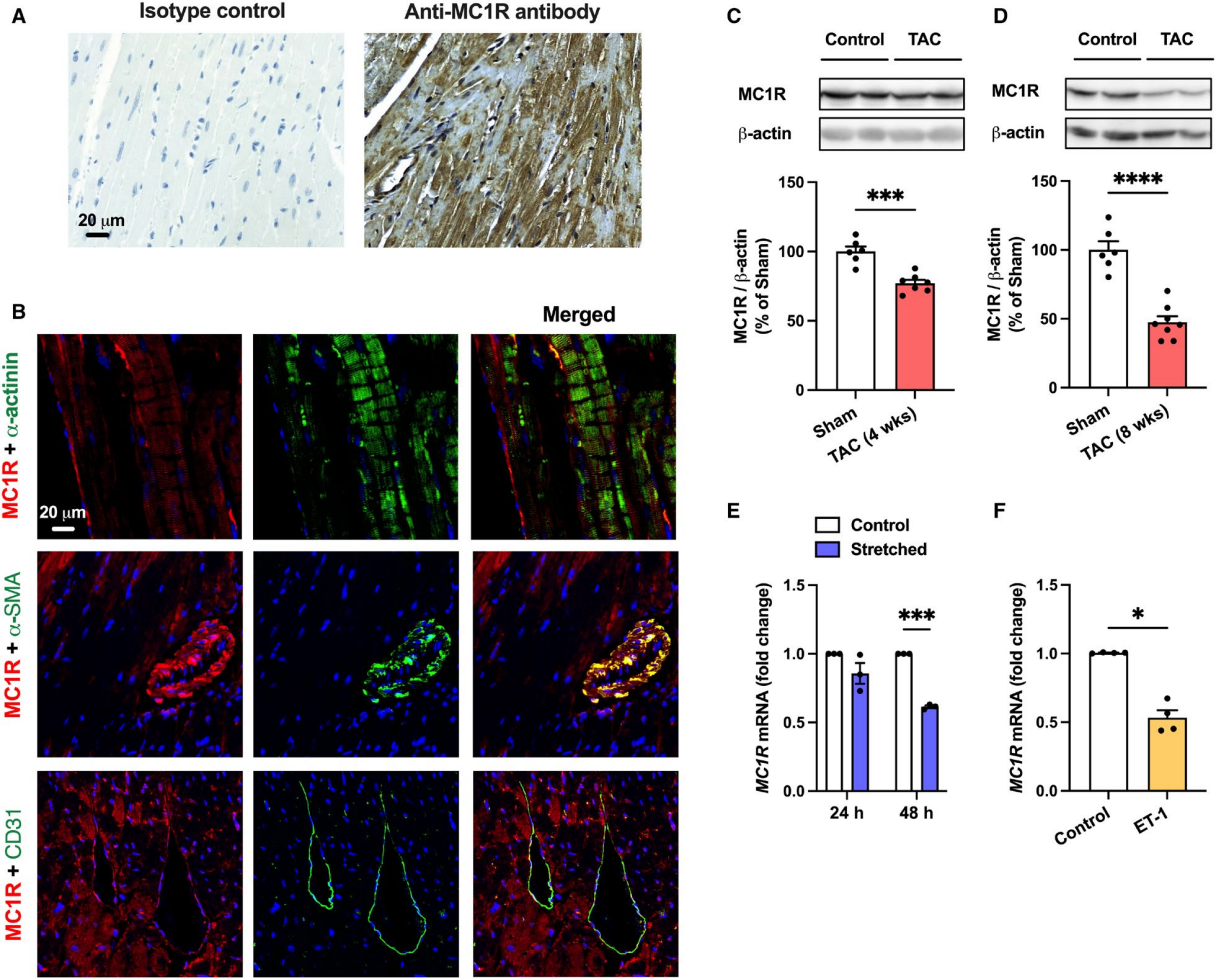
MC1R is expressed in the mouse heart and downregulated in pathological cardiac hypertrophy. **A**, Immunostaining of MC1R in cardiac cross‐section of C57Bl/6J mouse. In THE control section, anti‐MC1R antibody was replaced by purified normal rabbit IgG (isotype control). Scale bar 20 μm. **B**, Immunofluorescence staining of MC1R (red) and sarcomeric α‐actinin, α‐SMA or CD31 (green) in cardiac cross‐section of C57Bl/6J mouse. Scale bar 20 μm. **C** and **D**, Representative western blots and quantification of MC1R protein expression in the left ventricle of C57Bl/6J mice subjected to TAC for 4 or 8 wks. n=6–7 mice per group. ***P<0.001 and ****P<0.0001 vs sham by unpaired Student's *t* test. **E** and **F**, Quantitative real‐time polymerase chain reaction analysis of *MC1R* mRNA expression (normalized to the geometric mean of *GAPDH* and *RPS18*) in human induced pluripotent stem cell‐derived cardiomyocytes that were mechanically stretched for 24 or 48 h or treated with ET‐1 (100 nM) for 24 h. n=3–4 individual experiments/batches of differentiation. *P<0.05 and **P<0.01 vs control by 2‐way ANOVA and Šídák's post hoc test (**E**) or Mann–Whitney *U* test (**F**). Data are mean±SEM. α‐SMA indicates α‐smooth muscle actin; ET‐1, endothelin 1; MC1R, melanocortin 1 receptor; and TAC, transverse aortic constriction.

We first aimed to investigate whether MC1R is expressed in the adult mouse heart. Immunohistochemical staining of a cross‐section of the mouse heart showed a strong positive signal for MC1R (Figure 1A). Double immunofluorescence staining further revealed that MC1R expression is primarily localized to sarcomeric α‐actinin–expressing cardiomyocytes (Figure 1B). However, MC1R appeared to be present also in other cell types, since MC1R‐positive staining was detected in α‐smooth muscle actin–expressing smooth muscle cells and CD31‐expressing endothelial cells (Figure 1B).

We next explored whether the expression of MC1R in the heart is modulated during the development of pathological cardiac hypertrophy. To this end, we subjected mice to TAC‐induced pressure overload for 4 or 8 weeks and quantified MC1R protein expression in the left ventricle by Western blotting. The level of MC1R protein was significantly reduced in TAC‐operated mice after 4 weeks (Figure 1C), and it progressively declined toward a more advanced stage of pathological hypertrophy and heart failure (8 weeks after TAC; Figure 1D). A similar expression pattern was also observed in mice subjected to angiotensin II infusion, which was used as another model of pathological cardiac hypertrophy. Cardiac MC1R expression was not changed after 2 weeks of angiotensin II infusion (Figure S2A), while a 4‐week infusion of angiotensin II significantly reduced MC1R protein expression in the left ventricle (Figure S2B). Finally, we investigated whether *MC1R* is expressed in human cardiomyocytes and whether its expression is modulated by hypertrophic stimuli. hiPSC‐CMs showed gradual downregulation of MC1R after 24‐ or 48‐hour mechanical stretching (Figure 1E). Treatment of hiPSC‐CMs with endothelin 1, as another model of cardiomyocyte hypertrophy, also downregulated *MC1R* (Figure 1F). Collectively, these results demonstrate that MC1R is expressed in the mouse heart as well as in human cardiomyocytes, and its expression is modulated by different hypertrophic stimuli.

### Mice With Global MC1R Deficiency Show Blunted Response to Pathological or Physiological Cardiac Hypertrophy

**Figure 2:**
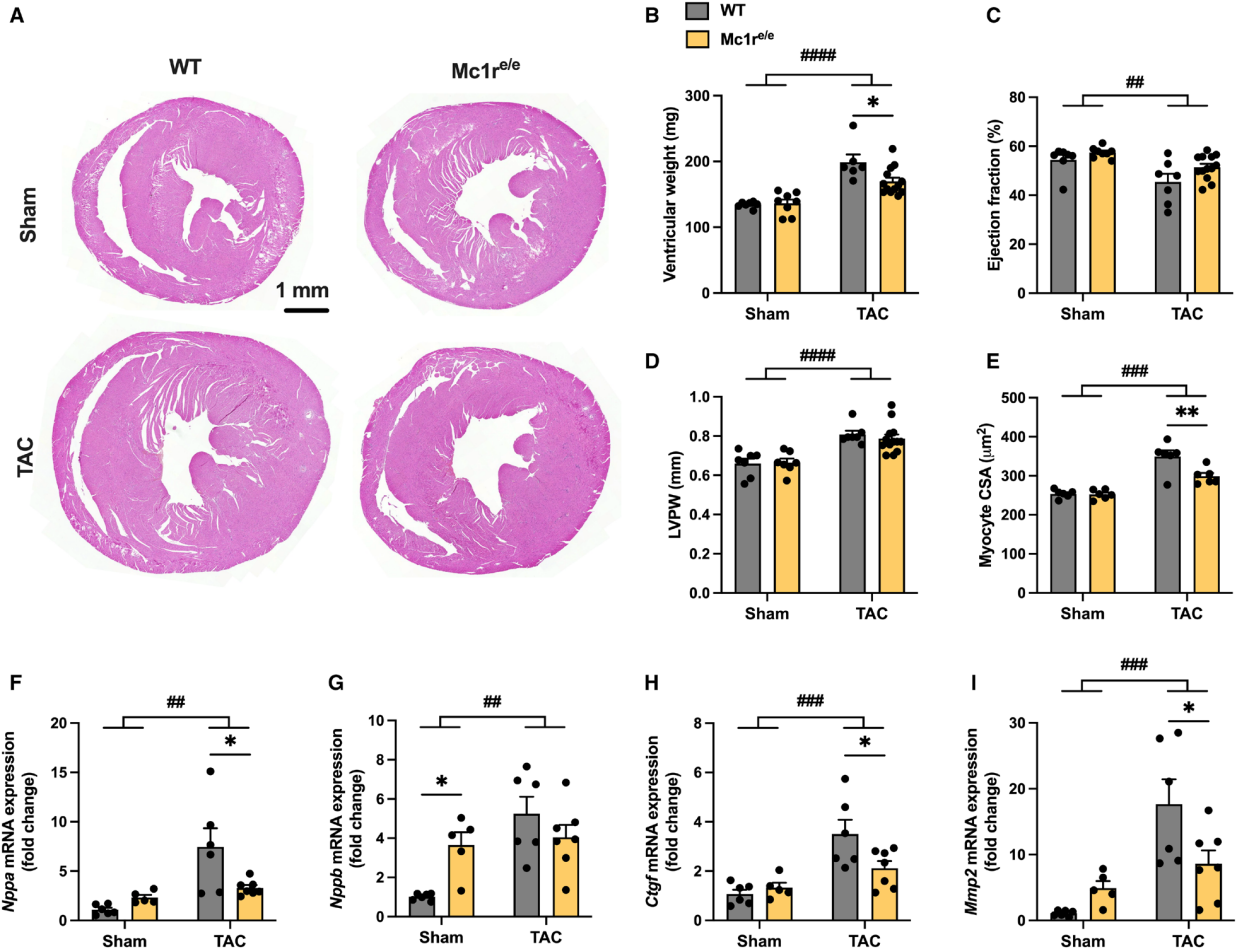
Global MC1R deficiency blunts pressure overload–induced cardiac hypertrophy. **A**, Representative hematoxylin and eosin–stained cross‐sections of the heart of sham‐ and TAC‐operated WT and Mc1r^e/e^ mice. Scale bar, 1 mm. **B**, Ventricular weight in WT and Mc1r^e/e^ mice at euthanasia after 8 wks of sham or TAC operation. **C** and **D**, Echocardiographic analysis of left ventricular ejection fraction and LVPW thickness. n=7 in sham WT, n=8 in sham Mc1r^e/e^, n=7 in TAC WT, and n=13 in TAC Mc1r^e/e^. **E**, Quantification of cardiomyocyte CSA in the left ventricle of sham‐ and TAC‐operated WT and Mc1r^e/e^ mice. n=6 mice per group. **F through I**, Quantitative real‐time polymerase chain reaction analysis of *Nppa* (atrial natriuretic peptide), *Nppb* (B‐type natriuretic peptide), *Ctgf* (connective tissue growth factor) and *Mmp2* (matrix metalloproteinase 2) in the left ventricle of indicated groups n=5 to 7 mice per group in each graph. Gene expression is normalized against the geometric mean of *Actb* and *Mrps18a*. Data are mean±SEM; each dot represents an individual mouse. *P<0.05 for the indicated comparisons by 2‐way ANOVA and Šídák's post hoc tests. ^##^ *P*<0.01, ^###^ *P*<0.001 and ^####^ *P*<0.0001 for the main effect of TAC by 2‐way ANOVA. CSA indicates cross‐sectional area; LVPW, left ventricular posterior wall; MC1R, melanocortin 1 receptor; Mc1r^e/e^, melanocortin 1 receptor recessive yellow; TAC, transverse aortic constriction; and WT, wild‐type.

The finding of pressure overload–induced reduction in ventricular MC1R expression prompted us to investigate whether MC1R has a regulatory role in pathological cardiac remodeling. For this purpose, we used global MC1R‐deficient mice (Mc1r^e/e^) and their age‐matched WT controls and subjected them first to 8 weeks of LV pressure overload by TAC surgery. At euthanasia, ventricular weight did not differ between sham‐operated WT and Mc1r^e/e^ mice (Figure 2A and 2B). However, Mc1r^e/e^ showed a blunted response to a TAC‐induced increase in ventricular weight (Figure 2A and 2B; Figure S3A and S3B). Echocardiography, performed at 8 weeks after surgery, revealed depressed systolic LV function in TAC‐operated mice, as evidenced by reduced LV ejection fraction (EF), but no genotype effect was noted in this regard (Figure 2C). MC1R deficiency did not significantly affect LV posterior wall thickness or LV end‐diastolic dimension (LVEDD) (Figure 2D; Figure S3C). However, histological analysis revealed reduced myocyte CSA in TAC‐operated Mc1r^e/e^ mice (Figure 2E). At the molecular level, Mc1r^e/e^ mice showed attenuation of TAC‐induced changes in the mRNA expression of hypertrophic markers. Specifically, cardiac *Nppa* expression was downregulated in TAC‐operated Mc1r^e/e^ mice compared with WT mice (Figure 2F). Similar trends were also observed in other markers of pathological cardiac hypertrophy such as *Ctgf* (connective tissue growth factor), *Mmp2* (matrix metalloproteinase‐2) and *Acta2* (α‐smooth muscle actin; *P*=0.080 by Šídák's post hoc test) (Figure 2H and 2I; Figure S3E). Interestingly, *Nppb* (B‐type natriuretic peptide) was upregulated in the LV of sham‐operated Mc1r^e/e^ mice, and it was not further increased after TAC operation (Figure 2G). Taken together, although only subtle changes were observed in absolute and relative ventricular weight, global MC1R deficiency attenuated the hypertrophic response to LV pressure overload at the cellular and molecular level.

Second, we investigated whether global MC1R deficiency also modulates the hypertrophic response to a physiological stimulus. WT and Mc1r^e/e^ mice were subjected to voluntary wheel running for 5 weeks and then phenotyped for cardiac structure and function. Monitoring of voluntary wheel running activity showed that mice gradually increased their daily running distance over the 5‐week period (Figure S4A and S4B). Importantly, cumulative or average daily running distance did not differ between the genotypes, indicating equal hypertrophic stimulus between the genotypes (Figure S4A and S4B). After 5 weeks of running, Mc1r^e/e^ mice had lower ventricular weight and ventricular weight‐to‐body weight ratio (Figure S4C and S4D). Myocyte CSA was also reduced in Mc1r^e/e^ mice (Figure S4E). Echocardiographic measurements of LV geometry revealed that LV posterior wall thickness increased from baseline to 5 weeks after exercise in WT mice, while no change of LV posterior wall was observed in Mc1r^e/e^ mice, indicating a lack of hypertrophic response (Figure S4F). LVEDD was unaffected by exercise training or MC1R deficiency (Figure S4G). Of note, MC1R deficiency dampened the exercise‐induced increase in LV systolic performance, as evidenced by lower EF in Mc1r^e/e^ mice compared with WT mice at the end of the experiment (Figure S4H). Collectively, these findings indicate that global MC1R deficiency attenuates both pathological and physiological cardiac remodeling.

### Cardiomyocyte‐Specific Deletion of MC1R Blunts Pathological Cardiac Hypertrophy

We next hypothesized that the phenotype of Mc1r^e/e^ mice is primarily driven by disturbed MC1R signaling in cardiomyocytes. To test this hypothesis, we engineered a tamoxifen‐inducible Mc1r‐cKO mouse model by crossing *MC1R* floxed mice with Myh6‐MCM transgenic mice. Efficient and specific Cre‐Lox recombination was verified by analyzing genomic DNA samples from the heart and different reference tissues at the end of the experiment (Figure S1).

**Figure 3:**
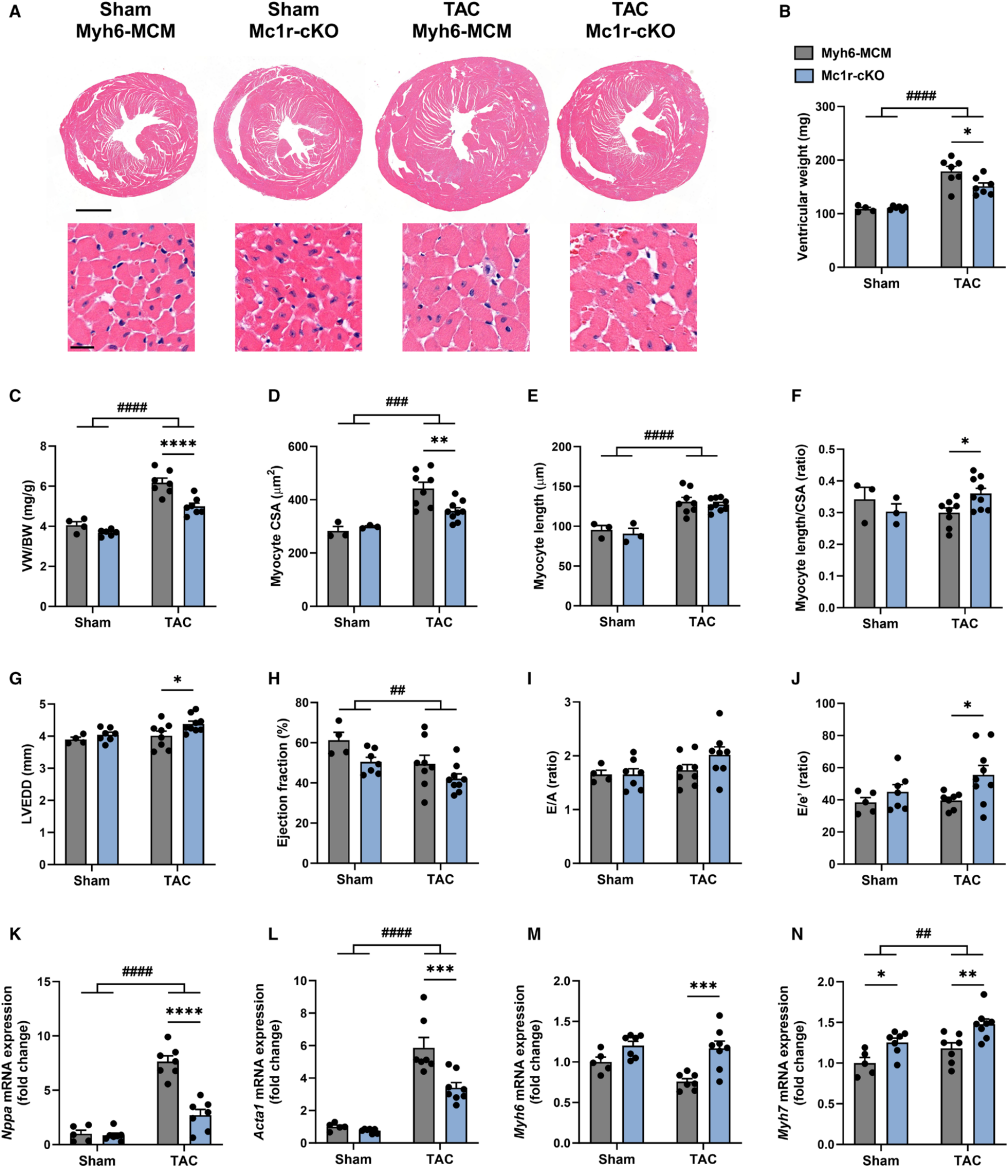
Cardiomyocyte‐specific MC1R knockout mice show reduced left ventricular hypertrophy and compromised cardiac function after TAC‐induced pressure overload. **A**, Representative hematoxylin and eosin–stained cross‐sections of the heart of sham‐ and TAC‐operated control (Myh6‐MCM) and Mc1r‐cKO mice. Scale bar: 1 mm (upper) and 20 μm (lower panel). **B** and **C**, Ventricular weight and VW/BW. **D through F**, Quantification of cardiomyocyte CSA, length and length‐to‐CSA ratio in the LV. **G through J**, Echocardiographic analyses of LVEDD and ejection fraction as well as mitral valve E/A ratio and E/e′ ratio in Myh6‐MCM and Mc1r‐cKO after 8 wks of sham or TAC operation. **K through N**, Quantitative real‐time polymerase chain reaction analysis of *Nppa*, *Acta1* (Actin, α‐skeletal muscle), *Myh6* (myosin heavy chain‐α) and *Myh7* (myosin heavy chain‐β) in the left ventricle of indicated groups. Gene expression is normalized against the geometric mean of *Actb* and *Mrps18a*. Data are mean±SEM; each dot represents an individual mouse. n=3–5 in sham Myh6‐MCM mice, n=3–7 in sham Mc1r‐cKO mice, n=7–8 in TAC Myh6‐MCM mice, and n=7–9 in TAC Mc1r‐cKO mice. *P<0.05, **P<0.01, ***P<0.001 and ****P<0.001 for the indicated comparisons by 2‐way ANOVA and Šídák's post hoc tests. ^##^ *P*<0.01, ^###^ *P*<0.001 and ^####^ *P*<0.0001 for the main effect of TAC by 2‐way ANOVA. CSA indicates cross‐sectional area; LVEDD, left ventricular end‐diastolic dimension; MC1R, melanocortin 1 receptor; Mc1r‐cKO, cardiomyocyte‐specific melanocortin 1 receptor knockout; Myh6‐MCM, Myh6‐MerCreMer transgenic; TAC, transverse aortic constriction; and VW/BW, ventricular weight‐to‐body weight ratio.

First, we subjected Mc1r‐cKO and Myh6‐MCM control mice to TAC or sham operation. After 8 weeks, TAC‐operated Mc1r‐cKO mice showed milder hypertrophic remodeling compared with Myh6‐MCM mice (Figure 3A), as evidenced by reduced ventricular weight (Figure 3B), ventricular weight‐to‐body weight ratio (Figure 3C), and ventricular weight‐to‐tibia length ratio (Figure S5A). Attenuated hypertrophic response of Mc1r‐cKO mice was also demonstrated by reduced myocyte CSA in the left ventricle (Figure 3D). However, length of ventricular cardiomyocytes was equally increased in TAC‐operated Myh6‐MCM and Mc1r‐cKO mice (Figure 3E). Consequently, myocyte length‐to‐CSA ratio was significantly higher in TAC‐operated Mc1r‐cKO mice (Figure 3F). These mice also displayed increased LVEDD, LV end‐diastolic volume, and LV end‐systolic volume (Figure 3G; Table S5), as measured by echocardiography at the end of the experiment. Furthermore, echocardiographic analyses showed a general reduction in EF in Mc1r‐cKO mice (Figure 3H; *P*=0.015 for genotype effect by 2‐way ANOVA), but post hoc comparisons between the genotypes did not reach statistical significance in sham‐ or TAC‐operated mice. However, stroke volume or cardiac output was not affected by cardiomyocyte‐specific MC1R deficiency (Table S5). Speckle tracking–based strain imaging revealed reduced global radial strain rate in sham‐ and TAC‐operated Mc1r‐cKO mice, while global longitudinal and circumferential strain and strain rate remained unchanged in these mice (Figure S6). Finally, lung weight was not affected by either TAC operation or genotype (Figure S5B), indicating that the mice had not developed pulmonary edema as a sign of congestive heart failure.

In terms of diastolic function, TAC‐operated Mc1r‐cKO mice showed a tendency (*P*=0.086) towards increased mitral valve E/A ratio (Figure 3I) as well as significantly increased E/e′ ratio (Figure 3J) and reduced isovolumetric relaxation time (Table S5). However, echocardiographic measurement of left atrial diameter revealed no significant differences between the genotypes (Table S5). Together, these results suggest increased LV filling pressure in TAC‐operated Mc1r‐cKO mice, but not necessarily diastolic dysfunction.

Corroborating the finding of reduced ventricular weight, gene expression analyses by qPCR revealed suppressed upregulation of hypertrophy‐related genes such as *Nppa* and *Acta1* in the left ventricle of TAC‐operated Mc1r‐cKO mice (Figure 3K and 3L), while *Nppb was* similarly upregulated in both genotypes after TAC operation (Figure S5C). Intriguingly, the mRNA levels of 2 forms of myosin heavy chain (MHC), α‐ and β (encoded by *Myh6* and *Myh7*, respectively), which are involved in cardiac contractility,^28^ were upregulated in Mc1r‐cKO mice (Figure 3M and 3N). These changes appeared in both sham‐ and TAC‐operated Mc1r‐cKO mice.

**Figure 4:**
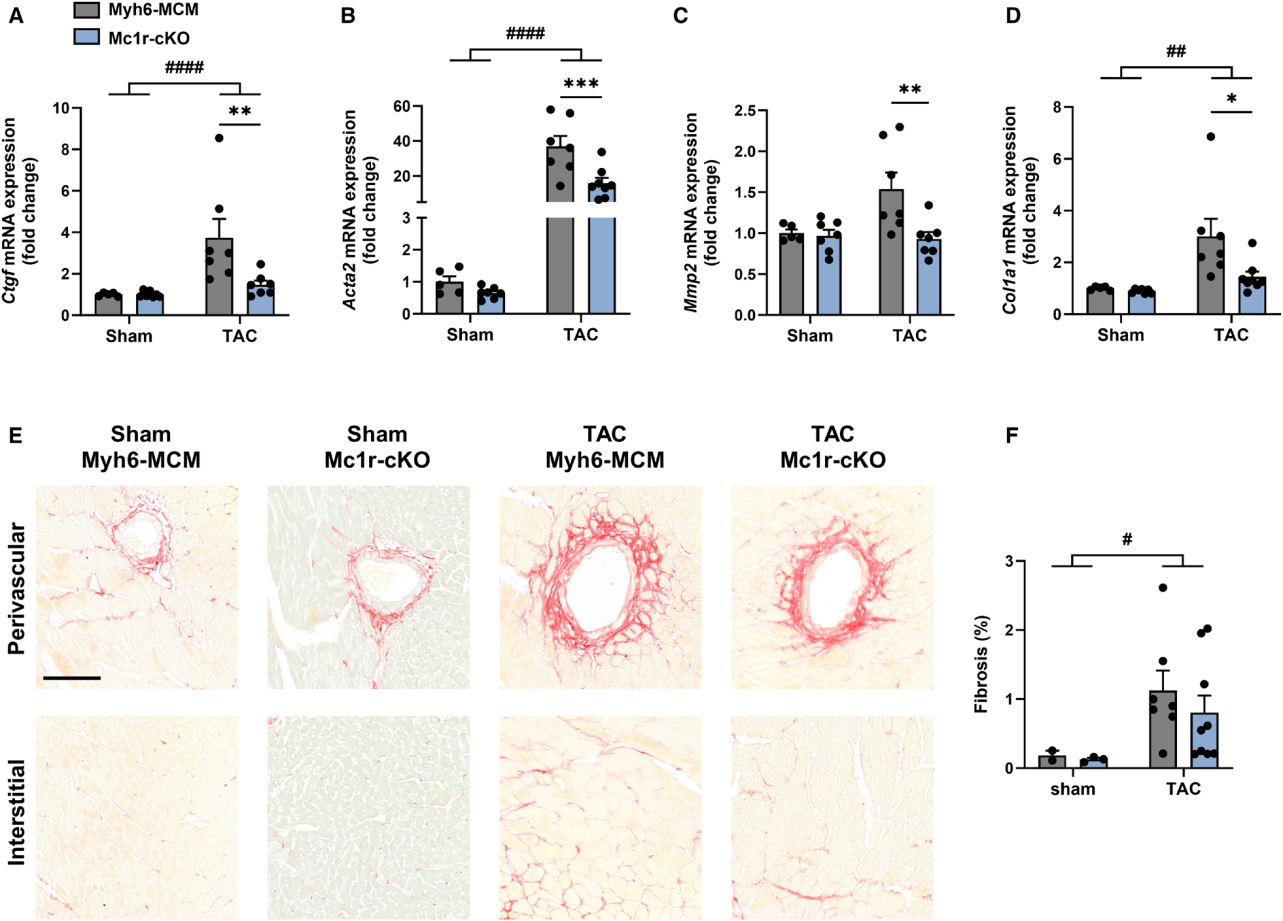
Cardiomyocyte‐specific deletion of MC1R does not affect cardiac fibrosis. **A through D**, Quantitative real‐time polymerase chain reaction analysis of *Ctgf*, *Acta2* (α‐smooth muscle actin), *Mmp2*, and *Col1a1* (collagen type I, α1) in the left ventricle of Myh6‐MCM and Mc1r‐cKO mice after 8 wks of sham or TAC operation. Gene expression is normalized against the geometric mean of *Actb* and *Mrps18a*. **E**, Representative Picrosirius Red–stained cardiac cross‐sections showing the extent of perivascular and interstitial fibrosis in the indicated groups. Scale bar, 100 μm. **F**, Quantification of fibrosis in the indicated groups. Data are mean±SEM; each dot represents an individual mouse. n=2–5 in sham Myh6‐MCM mice, n=3–7 in sham Mc1r‐cKO mice, n=7 in TAC Myh6‐MCM mice, and n=7–9 in TAC Mc1r‐cKO mice. *P<0.05, **P<0.01 and ***P<0.001 for the indicated comparisons by 2‐way ANOVA and Šídák's post hoc tests. ^#^ *P*<0.05, ^##^ *P*<0.01 and ^####^ *P*<0.0001 for the main effect of TAC by 2‐way ANOVA. MC1R indicates melanocortin 1 receptor; Mc1r‐cKO, cardiomyocyte‐specific melanocortin 1 receptor knockout; Myh6‐MCM, Myh6‐MerCreMer transgenic; and TAC, transverse aortic constriction.

The observed changes in LV systolic and diastolic function of Mc1r‐cKO mice raised a question whether MC1R deficiency promotes cardiac fibrosis, which is a hallmark of pathological cardiac remodeling and leads to stiffening of the myocardium. Quantification of cardiac mRNA levels of fibrosis‐related genes revealed significant downregulation of *Ctgf*, *Acta2*, *Mmp2*, and *Col1a1* in TAC‐operated Mc1r‐cKO mice compared with TAC‐operated control mice (Figure 4A through 4D). No change was observed in *Tgfb1* (transforming growth factor β1) or *Col3a1* (collagen type III, α1) mRNA levels (Figure S5D and S5E). Despite the changes in the molecular fingerprint of cardiac fibrosis, Picrosirius Red staining of cardiac cross‐sections did not show a parallel reduction in the extent of LV fibrosis in TAC‐operated Mc1r‐cKO mice (Figure 4E and 4F). Furthermore, the expression of proapoptotic markers such as *Casp3* (caspase‐3), *Bax* (BCL2‐associated X protein) and *Noxa* (phorbol‐12‐myristate‐13‐acetate‐induced protein 1) were unaffected by MC1R deficiency (Figure S5F through S5H).

**Figure 5:**
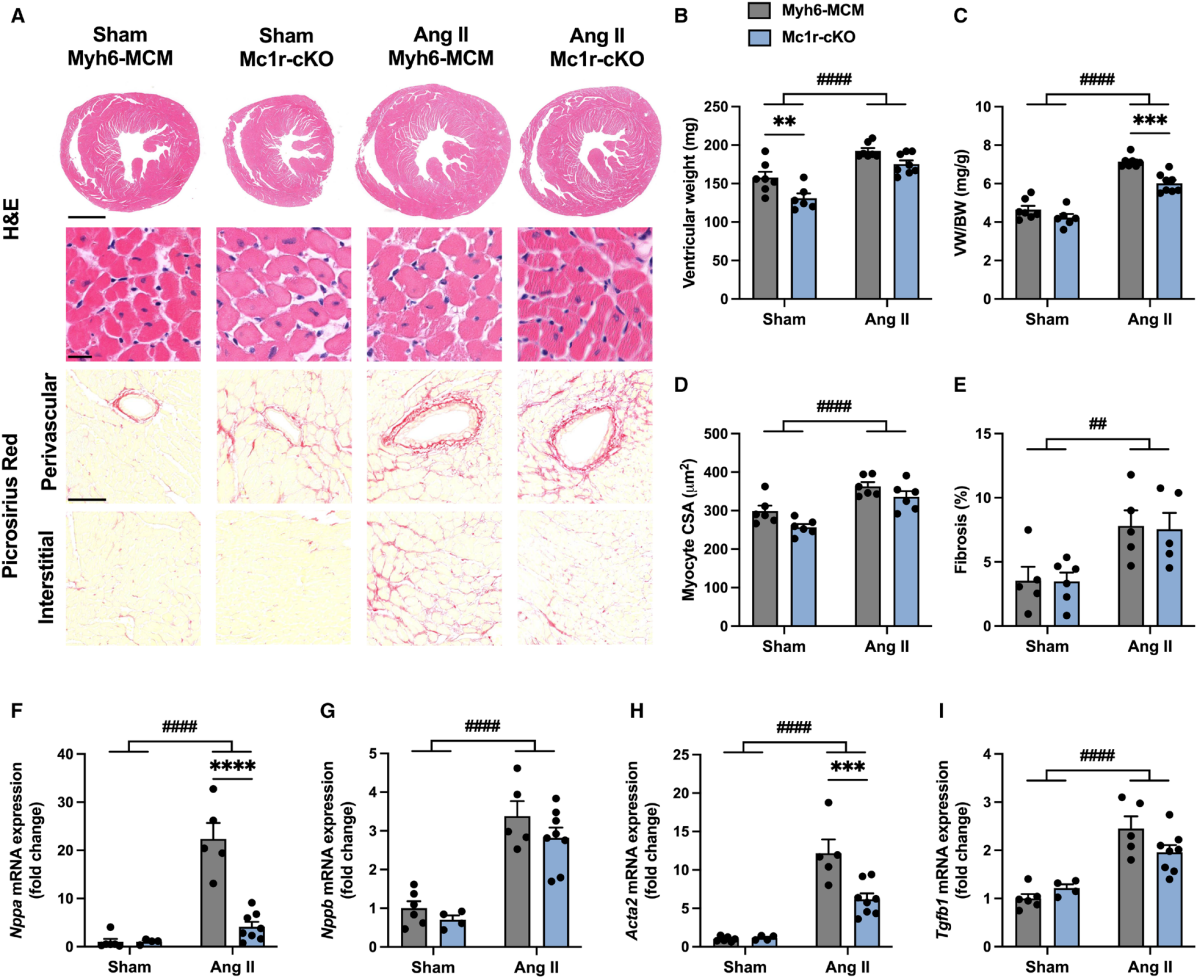
Cardiomyocyte‐specific MC1R knockout mice show reduced left ventricular hypertrophy after 4 wks of angiotensin II infusion. **A**, Representative H&E‐ and Picrosirius Red–stained cardiac cross‐sections in Myh6‐MCM and Mc1r‐cKO mice subjected to sham operation or 4 wks of Ang II infusion. Scale bars: 1 mm (upper) and 20 μm (lower panel) in H&E; 100 μm in Picrosirius Red. **B and C**, Ventricular weight and VW/BW ratio in Myh6‐MCM and Mc1r‐cKO mice at euthanasia after 4 weeks of angiotensin II infusion or sham operation. **D**, Quantification of cardiomyocyte CSA in the indicated groups. **E**, Quantification of the extent of left ventricular fibrosis in the indicated groups. **F through I**, Quantitative real‐time polymerase chain reaction analysis of *Nppa*, *Nppb*, *Acta2* and *Tgfb1* (transforming growth factor β) in the left ventricle of the indicated groups. Gene expression is normalized against the geometric mean of *Actb* and *Mrps18a*. Data are mean±SEM; each dot represents an individual mouse. n=5 to 7 in sham Myh6‐MCM mice, n=4 to 6 in sham Mc1r‐cKO mice, n=5 to 7 in angiotensin II Myh6‐MCM mice, and n=5 to 8 in angiotensin II Mc1r‐cKO mice. **P<0.01, ***P<0.001 and ****P<0.0001 for the indicated comparisons by 2‐way ANOVA and Šídák's post hoc tests. ^##^ *P*<0.01 and ^####^ *P*<0.0001 for the main effect of angiotensin II infusion by 2‐way ANOVA. Ang II indicates angiotensin II; CSA, cross‐sectional area; H&E, hematoxylin and eosin; MC1R, melanocortin 1 receptor; Mc1r‐cKO, cardiomyocyte‐specific melanocortin 1 receptor knockout; Myh6‐MCM, Myh6‐MerCreMer transgenic; TAC, transverse aortic constriction; and VW/BW, ventricular weight‐to‐body weight ratio.

Next, we aimed to test whether the phenotype of Mc1r‐cKO mice is independent of the chosen model of pathological cardiac hypertrophy. Thus, we subjected Mc1r‐cKO and Myh6‐MCM control mice to angiotensin II infusion for 4 weeks to introduce a different stimulus for pathological cardiac remodeling. Consistent with the TAC model, hematoxylin and eosin–stained cardiac cross‐sections and measurement of tissue weights at euthanasia showed blunted hypertrophic growth of the heart in angiotensin II–infused Mc1r‐cKO mice compared with Myh6‐MCM control mice (Figure 5A through 5C). This effect was most evident in ventricular weight‐to‐body weight ratio (Figure 5C). Absolute ventricular weight and ventricular weight‐to‐tibia length ratio were also reduced in sham‐operated Mc1r‐cKO mice (Figure 5B; Figure S7A). Angiotensin II infusion resulted in a similar increase in cardiomyocyte CSA in both genotypes (Figure 5D). However, Mc1r‐cKO mice displayed a general reduction in myocyte CSA (*P*=0.011 for genotype effect by 2‐way ANOVA). LV fibrosis was significantly enhanced by angiotensin II infusion, but no genotype effect was noted in this regard (Figure 5A and 5E). Additionally, qPCR analysis showed upregulation of the hypertrophy‐related genes *Nppa* and *Nppb* after the 4‐week angiotensin II infusion (Figure 5F and 5G) and Mc1r‐cKO mice were exclusively devoid of the induction of *Nppa*, which closely mirrors the gene expression profile observed in TAC‐operated Mc1r‐cKO mice. Likewise, the expression of the fibrotic gene *Acta2* was downregulated in angiotensin II–infused Mc1r‐cKO mice compared with angiotensin II–infused Myh6‐MCM mice (Figure 5H), and similar but nonsignificant trends were also observed in other fibrosis‐related genes such as *Tgfb1*, *Ctgf*, and *Mmp2* (Figure 5I, Figure S7B and S7C). Taken together, these findings demonstrate that MC1R deficiency in cardiomyocytes blunts pathological cardiac hypertrophy but leads to compromised systolic and diastolic function.

### Cardiac‐Specific MC1R Deficiency Causes a Functional Deficit in Response to Aerobic Exercise

**Figure 6:**
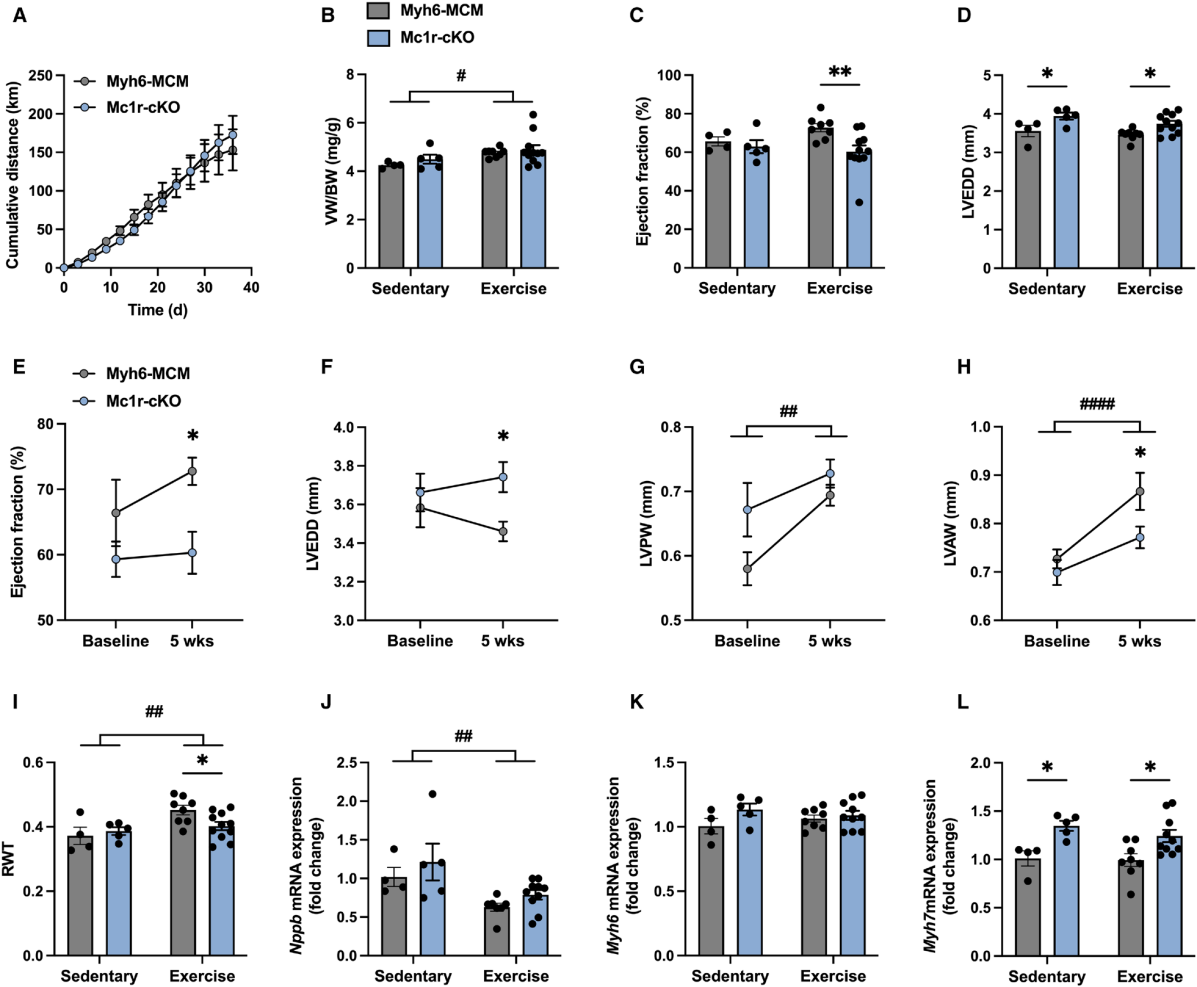
Cardiomyocyte‐specific deletion of MC1R causes insufficient left ventricular remodeling in response to voluntary wheel running. **A**, Cumulative running distance of Myh6‐MCM and Mc1r‐cKO mice during the 5‐wk running wheel experiment. The data were analyzed using 2‐way repeated measures ANOVA, *P*=0.88 for genotype effect. **B**, Ventricular weight in Myh6‐MCM and Mc1r‐cKO mice at euthanasia after the 5‐wk voluntary running (exercise group) or nonexercising period (sedentary group). **C** and **D**, Echocardiographic analysis of ejection fraction and LVEDD at the end of the experiment. **E through H**, Echocardiographic analysis of ejection fraction, LVEDD, LVPW thickness, and LVAW thickness at baseline and after 5 wks of voluntary wheel running. **I**, RWT at the end of the experiment. **J through L**, Quantitative real‐time polymerase chain reaction analysis of *Nppb*, *Myh6* and *Myh7* in the left ventricle of the indicated groups. Gene expression is normalized against the geometric mean of *Actb* and *Mrps18a*. Data are mean±SEM; each dot represents an individual mouse. n=4 in sedentary/Myh6‐MCM mice, n=5 in sedentary/Mc1r‐cKO mice, n=8 in exercise/Myh6‐MCM mice, and n=11 in exercise/Mc1r‐cKO mice. *P<0.05, **P<0.01 and ***P<0.001 for the indicated comparisons by 2‐way ANOVA and Šídák's post hoc tests. ^#^ *P*<0.05, ^##^ *P*<0.01 and ^####^ *P*<0.0001 for the main effect of exercise by 2‐way ANOVA **(B–D**, **I–L**). *P<0.05 vs Myh6‐MCM mice at 5 wks by 2‐way repeated measures ANOVA and Šídák's post hoc test (**E–H**). LVAW indicates left ventricular anterior wall; LVEDD, left ventricular end‐diastolic dimension; LVPW, left ventricular posterior wall; MC1R, melanocortin 1 receptor; Mc1r‐cKO, cardiomyocyte‐specific melanocortin 1 receptor knockout; Myh6‐MCM, Myh6‐MerCreMer transgenic; and RWT, relative wall thickness.

Finally, to evaluate whether cardiac‐specific MC1R deficiency also modulates structural or functional response to aerobic exercise, we subjected Myh6‐MCM and Mc1r‐cKO mice to the same 5‐week voluntary running protocol as Mc1r^e/e^ mice. Cumulative and average daily running distance was similar between control Myh6‐MCM and Mc1r‐cKO mice (Figure 6A; Figure S8A), indicating equal stimulus for physiological cardiac hypertrophy. Ventricular weight‐to‐body weight ratio was increased in the exercised group compared with the sedentary, nonrunning group (Figure 6B), but there were no significant differences between the genotypes in absolute or relative ventricular weights (Figure 6B; Figure S8C and S8D). Echocardiography at the end of the experiment showed lower EF in exercising Mc1r‐cKO mice compared with Myh6‐MCM mice (Figure 6C), while LVEDD was increased in both sedentary and exercising Mc1r‐cKO mice (Figure 6D). Assessment of structural and functional changes from baseline to 5 weeks after exercise further revealed that Mc1r‐cKO mice lacked the exercise‐induced improvement of EF (Figure 6E). Furthermore, the change in LVEDD was going to opposite directions between the genotypes (Figure 6F), and the exercise‐induced increase in LV posterior wall and LV anterior wall thickness was less pronounced in Mc1r‐cKO mice (Figure 6G and 6H). The respective changes from baseline to 5‐week time point in sedentary mice are presented in Figure S8E through S8H. Overall, the exercise‐induced changes in LV geometry were best reflected on the relative wall thickness, which was increased in the exercising Myh6‐MCM mice but not in Mc1r‐cKO mice (Figure 6I). This finding suggests that cardiomyocyte‐specific MC1R deficiency attenuated concentric LV hypertrophy in response to aerobic exercise.

Finally, we performed qPCR analysis of hypertrophy‐ and fibrosis‐related genes for LV samples from sedentary and exercise groups. Aerobic exercise did not affect cardiac *Nppa* expression (Figure S8I) but downregulated *Nppb* expression (Figure 6J). Although the mRNA levels of *Nppa* and *Nppb* were unaltered in Mc1r‐cKO mice, *Myh7* was significantly upregulated in both sedentary and exercising Mc1r‐cKO mice (Figure 6L). Other genes such as *Myh6*, *Acta1*, *Acta2*, and *Ctgf* were unaffected by either exercise or genotype (Figure 6K; Figure S5J through S8L). Taken together, these findings are in good agreement with the phenotype of exercising Mc1r^e/e^ mice and suggest that MC1R deficiency in the heart blunts the hypertrophic effect of physical activity.

### MC1R Activation in Cultured Cardiomyocytes Induces Hypertrophic Signaling

**Figure 7:**
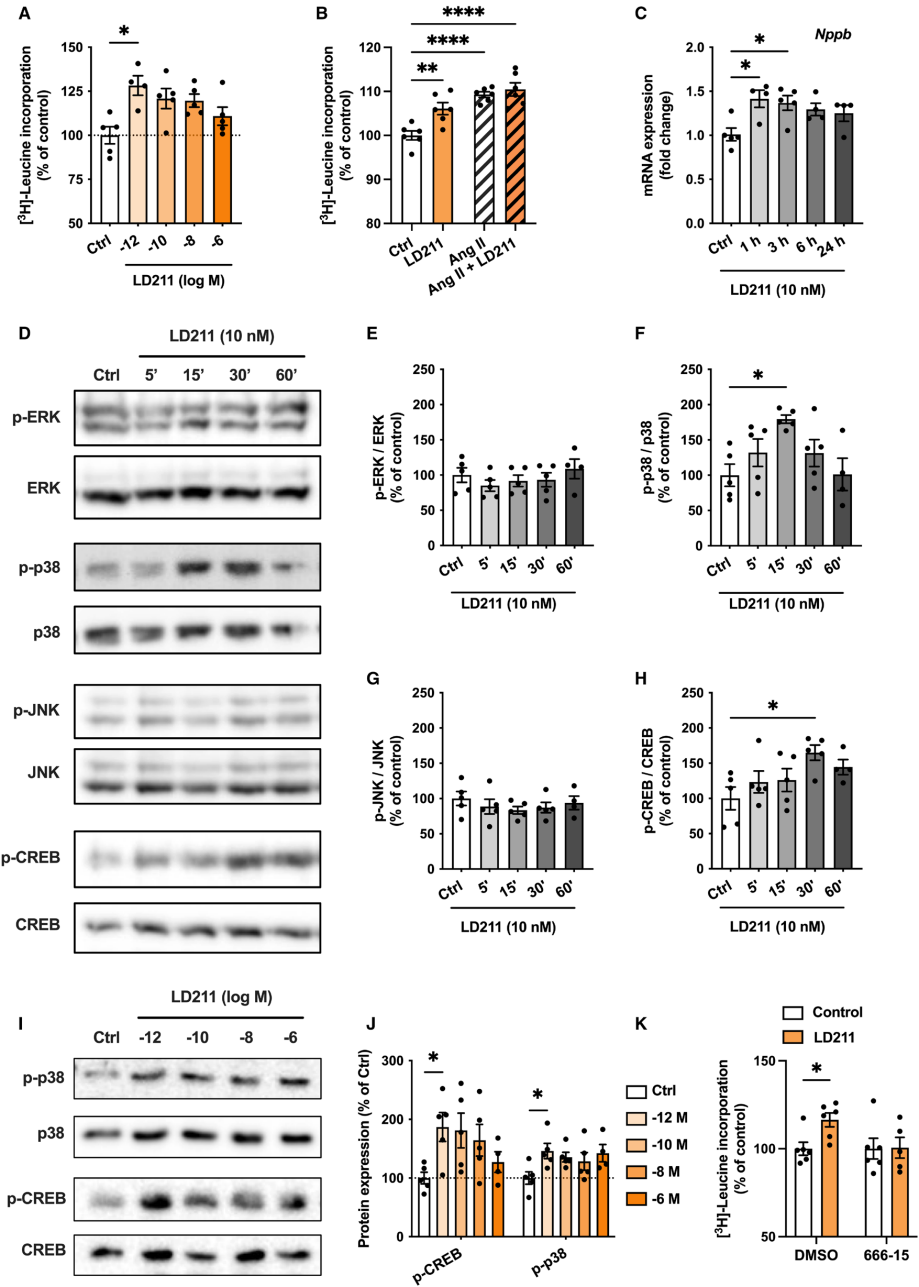
Pharmacological activation of MC1R promotes cardiomyocyte hypertrophy. **A**, [^3^H]‐Leucine incorporation assay in H9c2 cells treated with different concentrations of LD211 for 24 h. **B**, [^3^H]‐Leucine incorporation assay in H9c2 cells treated with LD211 (10 nM) or control (PBS) for 24 h in the absence or presence of angiotensin II (1 μM). **C**, Quantitative real‐time polymerase chain reaction analysis of *Nppb* mRNA expression in H9c2 cells treated with LD211 (10 nM) for 1, 3, 6 and 24 h. Gene expression is normalized against the geometric mean of *Gapdh* and *Rn18s*. **D through H**, Representative Western blots and quantification of p‐ERK, p‐p38, p‐JNK and p‐CREB in H9c2 cells treated with LD211 (10 nM) for 5, 15, 30, or 60 min. Data are expressed as a percentage of control. **I** and **J**, Representative western blots and quantification of p‐CREB and p‐p38 in H9c2 cells treated with different concentrations of LD211 for 15 min. Data are expressed as percentage of control. **K**, [^3^H]‐Leucine incorporation assay in H9c2 cells pretreated with or without the CREB inhibitor 666–15 (1 μM) for 2 h followed by treatment with LD211 (10 nM) or control (PBS) for 24 h. Data are mean±SEM, n=4–6 per group. *P<0.05, **P<0.01 and ****P<0.0001 for the indicated comparisons by 1‐way ANOVA and Dunnett's post hoc tests (**B**), Kruskal–Wallis and Dunn's post hoc tests (**A**, **C–J**) or 2‐way ANOVA and Šídák's post hoc tests (**K**). Ang II indicates angiotensin II; CREB, cAMP response element–binding protein; Ctrl, control; ERK, extracellular signal‐related kinase; JNK, c‐Jun N‐terminal kinase; and MC1R, melanocortin 1 receptor.

To investigate whether MC1R activation evokes the opposite phenotype compared with MC1R deficiency, we performed a set of in vitro experiments with H9c2 cells and NMCMs. First, we treated H9c2 cells with different concentrations of a selective MC1R agonist (LD211) for 24 hours and analyzed [^3^H]‐leucine incorporation as a measure of protein synthesis rate and hypertrophic growth. Activation of MC1R enhanced [^3^H]‐leucine incorporation with a stronger effect at lower concentrations of LD211 (Figure 7A). We also investigated whether LD211 amplifies angiotensin II–induced hypertrophic response. The hypertrophic effect of angiotensin II was slightly stronger compared with LD211 alone, but there was no further increase in [^3^H]‐leucine incorporation in cells treated with angiotensin II and LD211 (Figure 7B). To investigate whether activation of MC1R also induces changes at the transcriptional level, we treated H9c2 cells with LD211 for 1, 3, 6, and 24 hours and performed qPCR analysis, which revealed upregulation of the hypertrophy‐related gene *Nppb* (Figure 7C). We also explored the effects of LD211 on gene expression in NMCMs and observed that particularly fibrosis‐related genes, including *Tgfb1*, *Acta2*, and *Col1a1*, were upregulated in LD211‐treated cells (Figure S9A). These results demonstrate that pharmacological activation of MC1R induces a reverse phenotype compared with mice with global or cardiomyocyte‐specific MC1R deficiency.

Finally, we explored different intracellular signaling responses that could be evoked by selective MC1R activation. Since different MCR subtypes can be coupled to Gs proteins as well as to Gi proteins, we first measured cAMP levels in LD211‐treated H9c2 cells. However, LD211 did not change cAMP level under baseline conditions or in cells treated with the adenylyl cyclase activator forskolin (Figure S9B and S9C). Furthermore, no change was observed in intracellular Ca^2+^ levels after stimulation with LD211 (Figure S9D). Screening of other downstream targets of MC1R signaling revealed that LD211 increased phosphorylation of the mitogen‐activated protein kinase p38 as well as the phosphorylation of CREB with the peak effect appearing after 15 or 30 minutes of stimulation (Figure 7D, 7F and 7H). No change was observed in the phosphorylation of extracellular signal‐related kinases 1/2 or c‐Jun N‐terminal kinase (Figure 7D, 7E and 7G). In terms of p38 and CREB phosphorylation, the effect of LD211 was most potent at subnanomolar concentrations (Figure 7I and 7J). We then performed mechanistic experiments and first checked whether the LD211‐evoked hypertrophic response could be reversed by blocking the p38 pathway. The selective p38 inhibitor TAK‐715 reduced p38 phosphorylation but increased [^3^H]‐leucine incorporation (Figure S10A through S10C). Importantly, TAK‐715 did not block the increase in [^3^H]‐leucine incorporation in LD211‐treated cells (Figure S10C). However, in a similar experimental setup, the inhibition of CREB signaling with 666‐15 blocked the LD211‐induced increase in [^3^H]‐leucine incorporation (Figure 7K). Taken together, these results demonstrate that MC1R activation promotes cardiomyocyte hypertrophy and suggest a dependency of this effect on CREB signaling.

## Discussion

Melanocortin system and especially MC1R have been under intensive research for the past few decades and MC1R has proven to regulate a wide range of physiological functions from skin pigmentation to inflammation.^2^, ^9^ In the present study, we identified a novel regulatory role for MC1R in hypertrophic cardiac remodeling. We observed that MC1R is expressed in the mouse heart and downregulated in response to pathological cardiac hypertrophy. Global or cardiomyocyte‐specific silencing of MC1R signaling attenuated exercise‐ and pressure overload–induced cardiac hypertrophy but led concomitantly to LV dilatation and to subtle changes in LV systolic and diastolic function. Conversely, selective activation of MC1R promoted hypertrophy of cultured cardiomyocytes. Taken together, our results provide evidence that MC1R signaling in cardiomyocytes regulates physiological and pathological cardiac remodeling.

First, we demonstrated that MC1R is abundantly present in the mouse heart and that its expression gradually declines during the progression of pathological cardiac hypertrophy. Reduced MC1R expression in the heart could be a compensatory response to counteract pathological cardiac hypertrophy or result from altered levels of endogenous MC1R ligands. Immunostaining of MC1R in the mouse heart further revealed that MC1R localizes not only to cardiomyocytes but also to other major cell types including smooth muscle cells and endothelial cells. This was an expected finding, since previous studies have shown that functional MC1R exists in these cell types as well as in macrophages and fibroblasts.^9^, ^11^, ^20^, ^29^ However, despite showing expression in the rat heart and neonatal mouse heart, previous studies have not unequivocally proven that MC1R exists also in cardiomyocytes. The finding of downregulated *MC1R* expression in hiPSC‐CMs after prolonged stretching and pharmacologically induced hypertrophy demonstrate that MC1R also exists in human cardiomyocytes.

Demonstrating a role for MC1R signaling in pathological cardiac remodeling, we observed that global MC1R deficiency reduced ventricular weight and cardiomyocyte CSA and downregulated the cardiac expression of hypertrophic and fibrotic genes in TAC‐challenged Mc1r^e/e^ mice. Given the wide expression of MC1R also in nonmyocytes such as endothelial cells, fibroblasts, or macrophages, the phenotype of Mc1r^e/e^ mice could be driven by MC1R deficiency in multiple cell types. For example, Mc1r^e/e^ mice have endothelial dysfunction and increased arterial stiffness and pulse pressure due to nonfunctional MC1R in endothelial cells,^8^ which could increase cardiac afterload and explain why sham‐operated Mc1r^e/e^ mice showed increased *Nppb* expression. On the other hand, MC1R signaling in fibroblasts reduces activation and proliferation of these cells as well as collagen synthesis,^30^ while MC1R deficiency promotes fibrogenesis.^31^ Consequently, MC1R deficiency in endothelial cells or fibroblasts is unlikely to explain the reduced heart weight and the antihypertrophic and antifibrotic gene expression profile in TAC‐operated Mc1r^e/e^ mice. These phenotypic traits were recapitulated in TAC‐operated Mc1r‐cKO mice, indicating that MC1R signaling, specifically in cardiomyocytes, regulates pathological cardiac hypertrophy. A similar phenotype was observed in angiotensin II–infused Mc1r‐cKO, demonstrating that blunting of pathological cardiac hypertrophy appears independent of the chosen model.

A striking feature of pressure overloaded Mc1r‐cKO mice was that, despite reduced ventricular weight, LV systolic function was not improved. Instead, cardiomyocyte‐specific MC1R deficiency led to enhanced LV dilatation and to abnormalities in LV systolic and diastolic function after TAC surgery. However, there were no signs of exaggerated cardiac fibrosis in Mc1r‐cKO mice, which could explain the functional consequences of MC1R deficiency. At the cellular level, we observed reduced cardiomyocyte CSA in TAC‐operated Mc1r‐cKO mice that was associated with an equal lengthening of cardiomyocytes compared with TAC‐operated control mice, thus resulting in increased length‐to‐CSA ratio. Increased cardiomyocyte length and length‐to‐width ratio are architectural hallmarks of LV dilatation and development of heart failure with reduced ejection fraction.^32^ In eccentric LV hypertrophy, increased distending stress on the walls of the left ventricle causes new sarcomeres to be added in series, while in concentric hypertrophy, new sarcomeres are added in parallel, which increases myocyte and LV thickness.^33^ In Mc1r‐cKO mice, myocyte lengthening occurs without an appropriate increase in CSA, which indicates a lack of concentric LV remodeling in the compensated phase of TAC‐induced pressure overload. This, in turn, could predispose Mc1r‐cKO mice to LV dysfunction.

Echocardiographic measurements showed reduced EF and radial strain rate in Mc1r‐cKO mice after 8 weeks of TAC surgery, but no change in stroke volume or lung weight, suggesting that cardiomyocyte‐specific MC1R deficiency does not predispose to congestive heart failure. Accordingly, taking into account the unchanged stroke volume, the reduction of EF in Mc1r‐cKO could be primarily driven by the increase in LV end‐diastolic volume, and thus, it does not necessarily indicate systolic dysfunction. In terms of diastolic function, TAC‐operated Mc1r‐cKO mice displayed increased E/e′ ratio and reduced isovolumetric relaxation time, which could indicate increased LV filling pressure during diastole.^34^ E/e′ ratio is among the most used parameters, when evaluating abnormal LV filling pressures and progression of diastolic dysfunction both in experimental studies and in clinical practice.^35^, ^36^ Chronically elevated LV filling pressure typically leads to left atrial dilatation, which is an early marker of diastolic dysfunction. However, left atrial diameter was unchanged in Mc1r‐cKO mice, suggesting that these mice had not developed diastolic dysfunction after 8 weeks of TAC surgery.^37^

TAC‐operated Mc1r‐cKO mice showed distinct gene expression profile characterized by a marked downregulation of *Nppa*, unaltered *Nppb* expression and upregulation of *Myh7*, which could be linked to the observed changes in LV geometry and cardiac function. Studies on mice have uncovered that genetic deletion of *Nppa* or its target receptor natriuretic peptide receptor A enhances cardiac hypertrophy and LV dilatation and deteriorates LV systolic function after TAC‐induced pressure overload,^37^, ^38^ while deletion of *Nppb* does not affect susceptibility for pathological cardiac hypertrophy.^39^ These results imply that downregulation of *Nppa* in Mc1r‐cKO could be a causative factor for the LV dilatation and compromised cardiac function of Mc1r‐cKO, or merely a molecular fingerprint of reduced ventricular weight. The latter option is supported by the finding that MC1R activation per se had no effect on *Nppa* expression in cultured NMCMs. Another explanation for the changes in LV geometry and performance could be also related to the consistent upregulation of MHC‐β (encoded by *Myh7* gene) in Mc1r‐cKO mice. Pathological hypertrophic stimuli typically induce MHC‐β expression with simultaneous downregulation of MHC‐α (encoded by the *Myh6* gene).^28^ Relative expression of MHC‐α and MHC‐β is species‐dependent and tightly controlled by developmental stage.^40^, ^41^ Nevertheless, a similar shift in their relative expression (ie MHC‐β increase and MHC‐α decrease) occurs in the failing human and mouse heart.^28^, ^42^ Experimental evidence shows that this shift is not merely an adaptive response to preserve energy, but it has a detrimental effect on cardiac function. Mice with transgenic overexpression of MHC‐β had mildly reduced systolic function at baseline, but this functional deficit became more evident after exercise training and under chronic mechanical or pharmacological cardiovascular stress.^43^ Overexpression of MHC‐β was also associated with mildly increased LVEDD. This phenotype closely resembles the one observed in Mc1r‐cKO mice, and therefore increased MHC‐β expression could be a causative factor, but further studies are warranted to address this possibility. Admittedly, there are additional, yet unidentified, mechanisms that are likely to contribute to the observed phenotype of Mc1r‐cKO, especially regarding the antihypertrophic effect of MC1R deficiency.

In addition to affecting pathological cardiac remodeling, global and cardiomyocyte‐specific MC1R deficiency attenuated physiological cardiac hypertrophy and improvement in EF in response to 5‐week voluntary wheel running. Physiological cardiac hypertrophy is adaptive and reversible in nature and associated with increased EF. In humans, aerobic exercise typically induces volume overload and leads to eccentric cardiac hypertrophy.^44^ In contrast, voluntary wheel running in mice increases LV mass by ≈10% to 15% and does not directly affect LVEDD,^44^, ^45^ but can, for example, prevent LV dilatation in models of myocardial infarction and dilated cardiomyopathy.^46^, ^47^, ^48^ Thus, it appears that concentric LV remodeling after 5‐week voluntary wheel running was defective in Mc1r‐cKO mice. Instead, there was a shift toward eccentric LV remodeling, as evidenced by increased LVEDD. Collectively, the lack of exercise‐induced cardiac hypertrophy and improvement in EF indicate that MC1R deficiency is not beneficial for physiological cardiac remodeling.

To gain further insight into the effects and underlying mechanisms of MC1R signaling in cardiomyocytes, we performed in vitro experiments with H9c2 cells and NMCMs and found that selective MC1R activation promoted cellular growth. In terms of intracellular signaling, MC1‐R activation induced phosphorylation of p38 and CREB without a change in cAMP level. Intriguingly, we recently found that MC5R is also expressed in cardiomyocytes, where it mediates antihypertrophic regulation upon activation by its cognate ligand α‐melanocyte‐stimulating hormone.^23^ These results suggest that MC1R might be a counteractive receptor for MC5R in the heart. It could also be speculated that the balance between MC1R and MC5R expression in the heart dictates how the endogenous agonist α‐melanocyte‐stimulating hormone affects cardiac remodeling.

### Study Limitations

First, Mc1r‐cKO mice showed increased E/e′ ratio and reduced isovolumetric relaxation time, which, in the case of TAC model and restrictive filling pattern, points to diastolic dysfunction. These data should be interpreted with caution, since additional measurements such as LV filling pressures through invasive catheterization would be required to truly validate diastolic dysfunction in these mice. Second, increased cardiomyocyte length‐to‐CSA ratio in Mc1r‐cKO should be interpreted with caution and verified by morphological measurements in freshly isolated cardiomyocytes. Third, although mechanistic experiments suggest a dependency between MC1R‐evoked CREB phosphorylation and cardiomyocyte hypertrophy, further investigation is clearly needed to obtain conclusive evidence on whether changes in CREB signaling contribute to the observed phenotype of Mc1r‐cKO mice.

## Conclusions

The present study uncovers a novel functional role for MC1R in cardiac remodeling. Cardiomyocyte‐specific MC1R deficiency attenuates physiological and pathological cardiac hypertrophy in mice, while pharmacological activation of MC1R promotes cardiomyocyte hypertrophy. MC1R deficiency, however, led to LV dilatation and functional abnormalities, which manifested as reduced EF and signs of compromised diastolic function. These results might have clinical relevance as *MC1R* is highly polymorphic in humans and several loss‐of‐function variants have been identified and shown to be associated with red hair phenotype as well as with increased arterial stiffness and endothelial dysfunction. Furthermore, analogues of naturally occurring α‐melanocyte‐stimulating hormone (Scenesse, Vyleesi, and Imcivree),^49^ which have agonistic activity at MC1R, have been recently approved for clinical use in different therapeutic areas. Therefore, it is important to take into consideration and evaluate the potential effects of these drugs on the heart.

## Sources of Funding

This work was financially supported by grants from the Research Council of Finland (grant 315351 to Dr Rinne, grant 321564 to Dr Talman, and grant 333284 to Dr Kerkelä), the Sigrid Jusélius Foundation (to Drs Rinne, Talman, and Kerkelä), the Finnish Cultural Foundation (to Dr Rinne, A. Suominen, and L. Pohjolainen), Drug Research Doctoral Programme (to A. Suominen), the Finnish Foundation for Cardiovascular Research (to A. Suominen and L. Pohjolainen and Drs Talman, Kerkelä, and Rinne), the Instrumentarium Science Foundation (to A. Suominen), and the Emil Aaltonen Foundation (to A. Suominen).

## Disclosures

None.

## Supporting information

Tables S1–S5
Figures S1–S10
