# Supplementary material for: Melanocortin 1 Receptor Regulates Pathological and Physiological Cardiac Remodeling
Source: J Am Heart Assoc. 2025 Feb 8;14(4):e037961. doi: 10.1161/JAHA.124.037961 (PMC12074753; doi:10.1161/JAHA.124.037961)
Supplement: Supplementary file 1 — Tables S1–S5 Figures S1–S10 [file JAH3-14-e037961-s001.pdf]

Supplemental Material

Table S1: Mortality rates of $Mc1r^{e/e}$ and Mc1-cKO mice and their respective controls subjected to sham or TAC surgery

**Number of deaths**	Sham WT	Sham $Mc1r^{e/e}$	TAC WT	TAC $Mc1r^{e/e}$
Intra-/perioperative period (0-72 h)	1	1	3	5
Postoperative period (72 h - 8 wks)	-	-	2	1

**Number of deaths**	Sham Myh6-MCM	Sham Mc1r-cKO	TAC Myh6-MCM	TAC Mc1r-cKO
Intra-/perioperative period (0-72 h)	1	1	6	5
Postoperative period (72 h - 8 wks)	-	-	-	-

Table S2: Quantitative RT-PCR primers for mouse genes

Gene name (accession number)	5'-3' primer sequence
Acta1 (NM_001272041.1)	Fwd: cccaaagctaaccgggagaag
	Rev: ccagaatccaacacgatgcc
Acta2 (NM_007392.3)	Fwd: agattgtgcgcgacatcaaag
	Rev: gcagactccataccgataaagga
Actb (NM_007393.5)	Fwd: tccatcatgaagtgtgacgt
	Rev: gagcaatgatcttgatcttca
Bax (NM_007527.4)	Fwd: aaactggtgctcaaggcc
	Rev: cttggatccagacaagcagc
Casp3 (NM_009810.3)	Fwd: tggtgatgaaggggtcatttatg
	Rev: ttcggctttccagtcagactc
Col1a1 (NM_007742.4)	Fwd: gctcctcttaggggccact
	Rev: ccacgtctcaccattgggg
Col1a2 (NM_007743.3)	Fwd: tgcagtaacttcgtgcctagc
	Rev: acgtggtcctctgtctcca
Col3a1 (NM_009930.2)	Fwd: ctaaaattctgccaccccgaa
	Rev: aggatcaacccagtattctccactc
Ctgf (NM_010217.2)	Fwd: agacctgtgggatgggcat
	Rev: gcttggcgattttaggtgtcc
Fn1 (NM_010233.2)	Fwd: atgtggacccctcctgatagt
	Rev: gcccagtgatttcagcaaagg
Mc1r (NM_008559.3)	Fwd: gtgctggttgtgatagccatc
	Rev: tgctgacacttaccatcaggt
Mmp2 (NM_008610.3)	Fwd: gatgtcgcccctaaaacagac
	Rev: cagccatagaaagtgttcaggt
Myh6 (NM_010856.4)	Fwd: ccacttctccttggtccactatg
	Rev: acaaacccaccaccgtctca
Myh7 (NM_080728.3)	Fwd: agggtggcaaagtcactgct
	Rev: catcacctggtcctccttca
Noxa (NM_021451.2)	Fwd: gcagagctaccacctgagttc
	Rev: cttttgcgacttcccaggca
Nppa *(NM_008725.3)*	Fwd: gcttccaggccatattggag
	Rev: gggggcatgacctcatctt

Table S2 (continues): Quantitative RT-PCR primers for mouse genes

Gene name (accession number)	5'-3' primer sequence
Nppb (NM_008726.6)	Fwd: cccaaaaagagtccttcggtc
	Rev: cggtctatcttgtgcccaaag
Mrps18a (NM_026768.3)	Fwd: cagctccaagcgttcctgg
	Rev: ggccttcaattacagtcgtct
Serca2a (NM_001110140.3)	Fwd: gagaacgctcacacaaagacc
	Rev: cttcttcagccggcaattcgttg
Tgfb1 (NM_011577.2)	Fwd: ccgcaacaacgccatctatg
	Rev: cccgaatgtctgacgtattgaag

Table S3: Quantitative RT-PCR primers for rat genes

Gene name (accession number)	5'-3' primer sequence
Gapdh (NM_017008.4)	Fwd: gacatgccgcctggagaaac
	Rev: agcccaggatgccctttagt
Nppb (NM_031545.1)	Fwd: acaatccacgatgcagaagct
	Rev: gggccttggtcctttgaga
Rn18s (NR_046237.2)	Fwd: cattcgaacgtctgccctat
	Rev: gtttctcaggctccctctcc

Table S4: Quantitative RT-PCR primers for human genes

Gene name (accession number)	5'-3' primer sequence
GAPDH (NM_002046.7)	Fwd: tcaaggctgagaacgggaag
	Rev: cgccccacttgattttggag
MC1R (NM_002386.4)	Fwd: acacctggaggggaagaact
	Rev: aggaagcaggaaggagtcgt
RPS18 (NM_022551.3)	Fwd: cgccgctagaggtgaaattc
	Rev: ccagtcggcatcgtttatgg

Table S5: Echocardiography in sham- and TAC-operated Myh6-MCM and Mc1r-cKO mice at the end of the experiment (8 weeks after operation).

**Parameter**	**Sham Myh6-MCM** n=4	**Sham Mc1r-cKO** n=7	**TAC Myh6-MCM** n=8	**TAC Mc1r-cKO** n=9
**Transverse aorta diameter (mm)**	1.41 ± 0.02	1.40 ± 0.02	0.58 ± 0.01####	0.55 ± 0.01####
**Body weight (g)**	27.2 ± 0.9	30.0 ± 0.4*	28.8 ± 0.8	30.8 ± 0.6
**HR (bpm)**	340 ± 8	332 ± 11	363 ± 14	358 ± 13
**SV (µl)**	40.4 ± 2.5	37.7 ± 1.2	37.6 ± 1.6	36.6 ± 2.1
**CO (ml/min)**	13.5 ± 1.0	12.0 ± 0.9	12.7 ± 1.2	13.0 ± 1.1
**EDV (µl)**	66.0 ± 2.4	72.1 ± 3.0	71.6 ± 5.5#	87.2 ± 4.0*#
**ESV (µl)**	25.6 ± 2.4	35.9 ± 2.7	36.7 ± 4.3#	50.6 ± 3.4*#
**MV E (mm/s)**	635 ± 25	562 ± 20	653 ± 49	641 ± 45
**MV A (mm/s)**	326 ± 42	346 ± 18	381 ± 28	300 ± 26
**MV e' (mm/s)**	16.9 ± 1.3	12.9 ± 0.9*	15.5 ± 1.3	11.4 ± 1.4*
**IVRT/MV ET**	0.39 ± 0.02	0.39 ± 0.03	0.40 ± 0.03	0.31 ± 0.01*
**DT (ms)**	24.6 ± 5.1	21.7 ± 1.7	17.4 ± 2.9	28.4 ± 4.1
**LA diameter (mm)**	2.37 ± 0.05	2.71 ± 0.13	2.97 ± 0.12#	2.85 ± 0.08#

Data are mean ± SEM. * P<0.05 versus sham/TAC-operated Myh6-MCM mice, # P<0.05 and #### P<0.0001 for the main effect of TAC by 2-way ANOVA. HR indicates heart rate; SV, stroke volume; CO, cardiac output; EDV, end-diastolic volume; ESV, end-systolic volume; MV E, mitral valve E wave velocity; MV A, mitral valve A wave velocity; MV e', mitral annular e' wave velocity (tissue Doppler); IVRT/MV ET, interventricular relaxation time (IVRT) normalized by mitral valve ejection time (MV ET); DT, mitral valve E wave deceleration time; LA, left atrium.

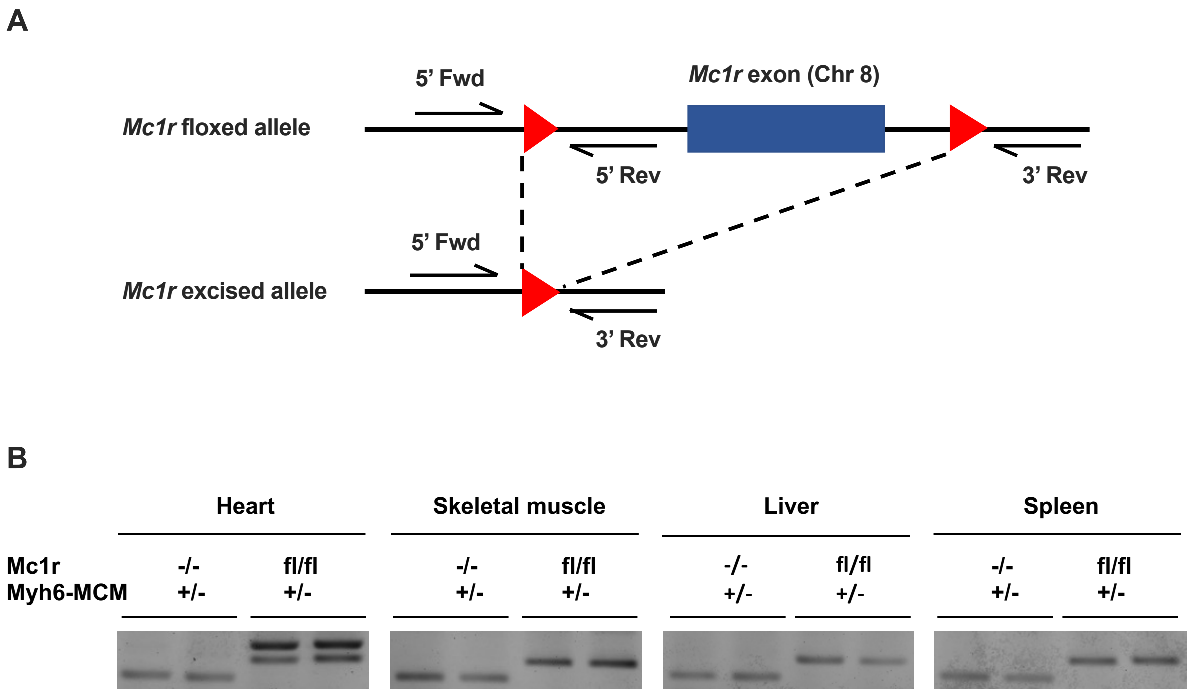

Figure S1. Generation of tamoxifen-inducible cardiomyocyte-specific MC1R knock-out (Mc1r-cKO) mice. (**A**) Schematic presentation of the loxP-flanked (floxed) *Mc1r* allele and the excised *Mc1r* allele after Myh6-MCM-mediated recombination. Fwd and Rev indicate forward and reverse primer positions used for PCR genotyping. Red arrow heads indicate loxP-sites. (**B**) PCR analysis of genomic DNA extracted from the heart, skeletal muscle (quadriceps femoris), liver and spleen of Cre-positive $Mc5^{wt/wt}$ mice and Cre-positive homozygous $Mc1r^{fl/fl}$ mice after 10 weeks of tamoxifen treatment. The PCR product of the recombined and excised *Mc1r* allele is slightly bigger than the $Mc1r^{fl/fl}$ allele and thus, appears on top of the $Mc1r^{fl/fl}$ allele in the heart samples but not in other tissues.

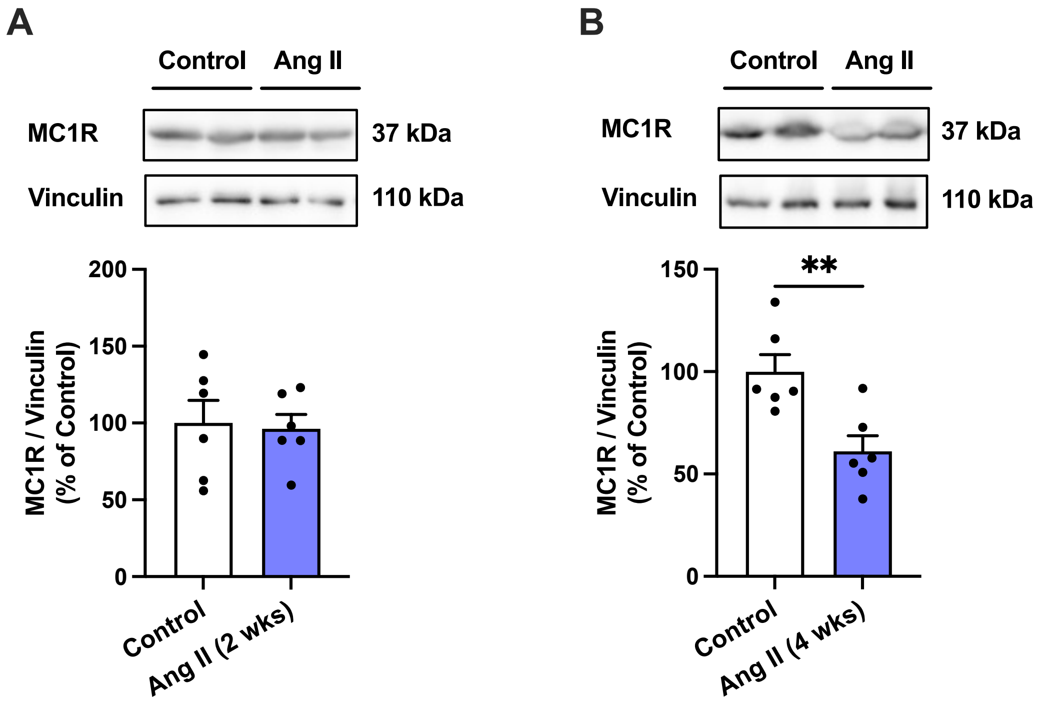

Figure S2. MC1R protein expression in the heart of Ang II-infused mice. (A) Representative Western blots and quantification of MC1R (normalized to vinculin) protein expression in the LV samples of control and Ang II-infused (2 weeks) mice. n=6 mice per group. **(B)** Representative Western blots and quantification of MC1R (normalized to vinculin) protein expression in the LV samples of control and Ang II-infused (4 weeks) mice. Data are mean ± SEM, each dot represents individual mouse. n=6 mice per group. ** P<0.01 versus control by unpaired Student's t test.

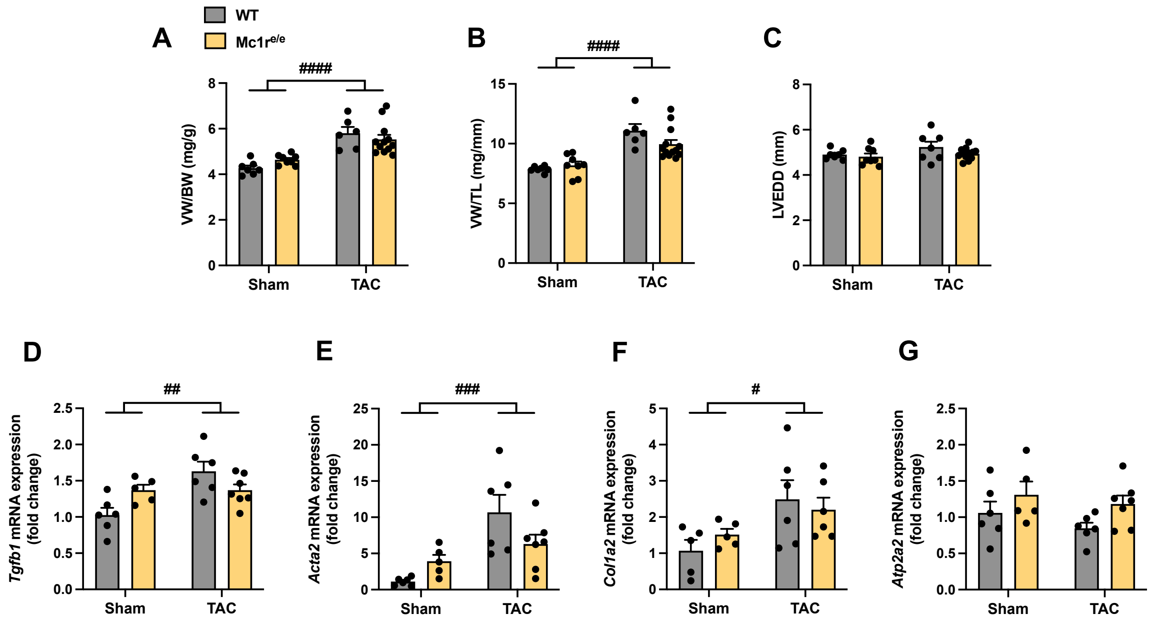

Figure S3. Ventricular weight, echocardiography and gene expression analysis in WT and Mc1r$^{e/e}$ mice after 8 weeks of sham or TAC operation. (**A**, **B**) Ventricular weight to body weight ratio (VW/BW) and ventricular weight to tibia length ratio (VW/TL) in WT and Mc1r$^{e/e}$ mice after 8 weeks of sham or TAC operation. (**C**) Echocardiographic analysis of LV end-diastolic diameter (LVEDD) in WT and Mc1r$^{e/e}$ mice after 8 weeks of sham or TAC operation. n=7 in sham WT mice, n=8 in sham Mc1r$^{e/e}$ mice, n=6 in TAC WT mice and n=13 in TAC Mc1r$^{e/e}$ mice. (**D**-**G**) qPCR analysis of *Tgfb1* (transforming growth factor beta 1), *Acta2* (alpha-smooth muscle actin), *Col1a2* (collagen type I, alpha 2) and *Atp2a2* (Sarcoplasmic/endoplasmic reticulum calcium ATPase 2/SERCA2) in the LV of WT and Mc1r$^{e/e}$ mice after sham or TAC surgery. Gene expression is normalized against the geometric mean of *Actb* and *Mrps18a*. n=5-6 mice per group in each graph. Data are mean ± SEM, each dot represents individual mouse. # $P<0.05$, ## $P<0.01$, ### $P<0.001$ for the main effect of TAC by 2-way ANOVA.

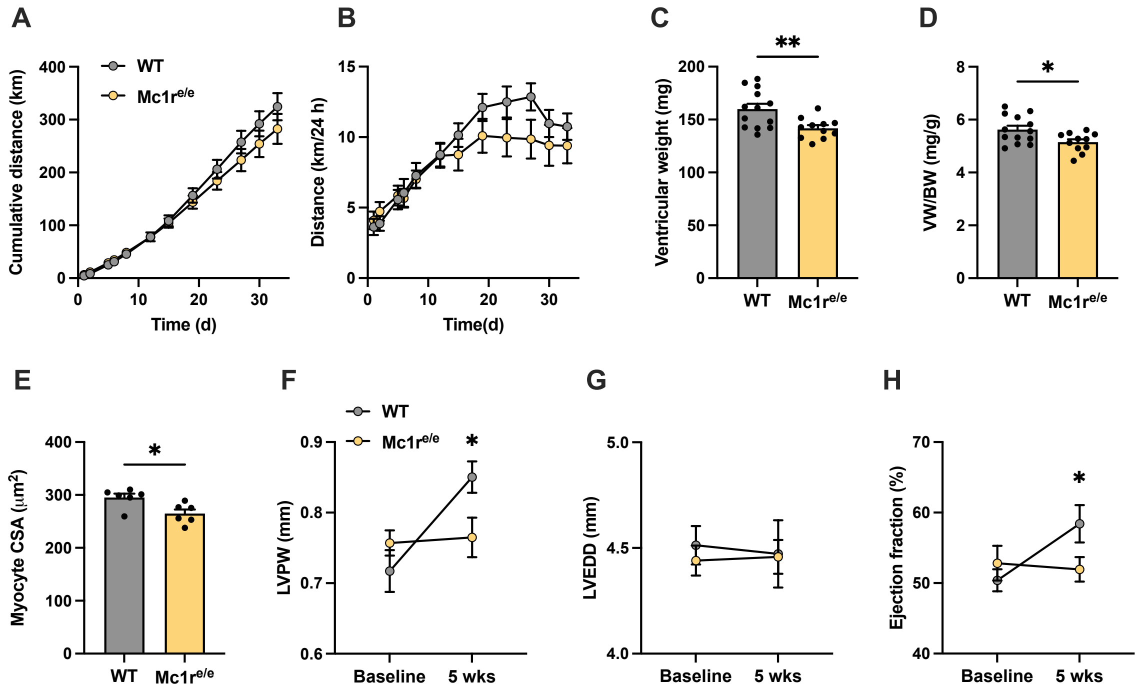

Figure S4. MC1R deficiency restrains physiological cardiac hypertrophy induced by voluntary wheel running. (**A and B**) Cumulative running distance and average 24-hour running distance in WT and Mc1r$^{e/e}$ mice subjected to voluntary wheel running for 5 weeks. The data was analyzed using 2-way repeated measures ANOVA, *P*=0.46 (**A**) and *P*=0.39 (**B**) for genotype effect. (**C, D**) Ventricular weight and ventricular weight to body weight ratio (VW/BW) in WT and Mc1r$^{e/e}$ mice after 5 weeks of voluntary wheel running. n=13 in WT mice, n=11 in sham Mc1r$^{e/e}$ mice. (**E**) Quantification of cardiomyocyte cross sectional area (CSA) in the LV of WT and Mc1$^{re/e}$ mice. n=6 mice per group. * *P*<0.05 and ** *P*<0.01 versus WT by unpaired Student's t test. (**F-H**) Echocardiographic analysis of LV posterior wall thickness (LVPW), LVEDD and LV ejection fraction in WT and Mc1r$^{e/e}$ mice at baseline and after 5 weeks of voluntary wheel running. n=10 mice per group. * *P*<0.05 versus WT using two-way repeated measures ANOVA and Šídák's *post hoc* test. Data are mean ± SEM, each dot represents individual mouse.

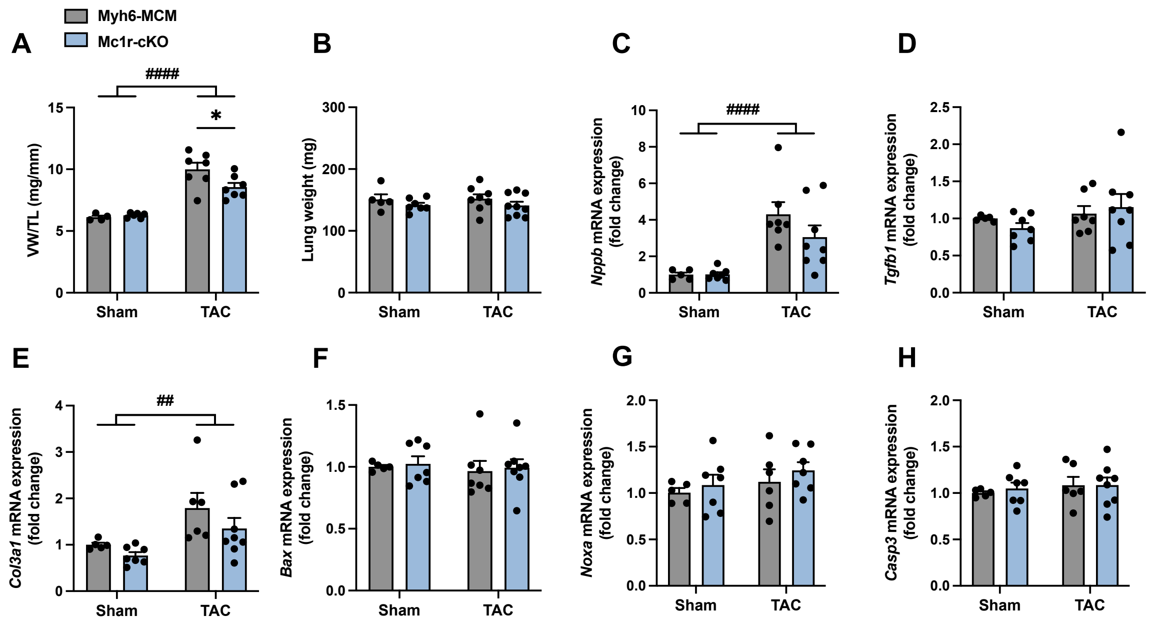

Figure S5. Cardiomyocyte-specific deletion of MC1R blunts TAC-induced pathological cardiac hypertrophy. (**A**) Ventricular weight to tibia length ratio (VW/TL) in Myh6-MCM control mice and Mc1r-cKO mice after 8 weeks of sham or TAC operation. (**B**) Lung weight at the end of the experiment. (**C**-**H**) qPCR analysis of *Nppb* (B-type natriuretic peptide), *Tgfb1*, *Col3a1* (collagen type III, alpha 1), *Casp3* (caspase-3), *Bax* (BCL2 associated X protein) and *Noxa* (phorbol-12-myristate-13-acetate-induced protein 1) in the LV of Myh6-MCM and Mc1r-cKO mice after sham or TAC surgery. Gene expression is normalized against the geometric mean of *Actb* and *Mrps18a*. n=5-8 mice per group in each graph. * $P<0.05$ for the indicated comparison by 2-way ANOVA and Šídák's *post hoc* test. ## $P<0.01$ and #### $P<0.0001$ for the main effect of TAC by 2-way ANOVA. Data are mean ± SEM, each dot represents individual mouse.

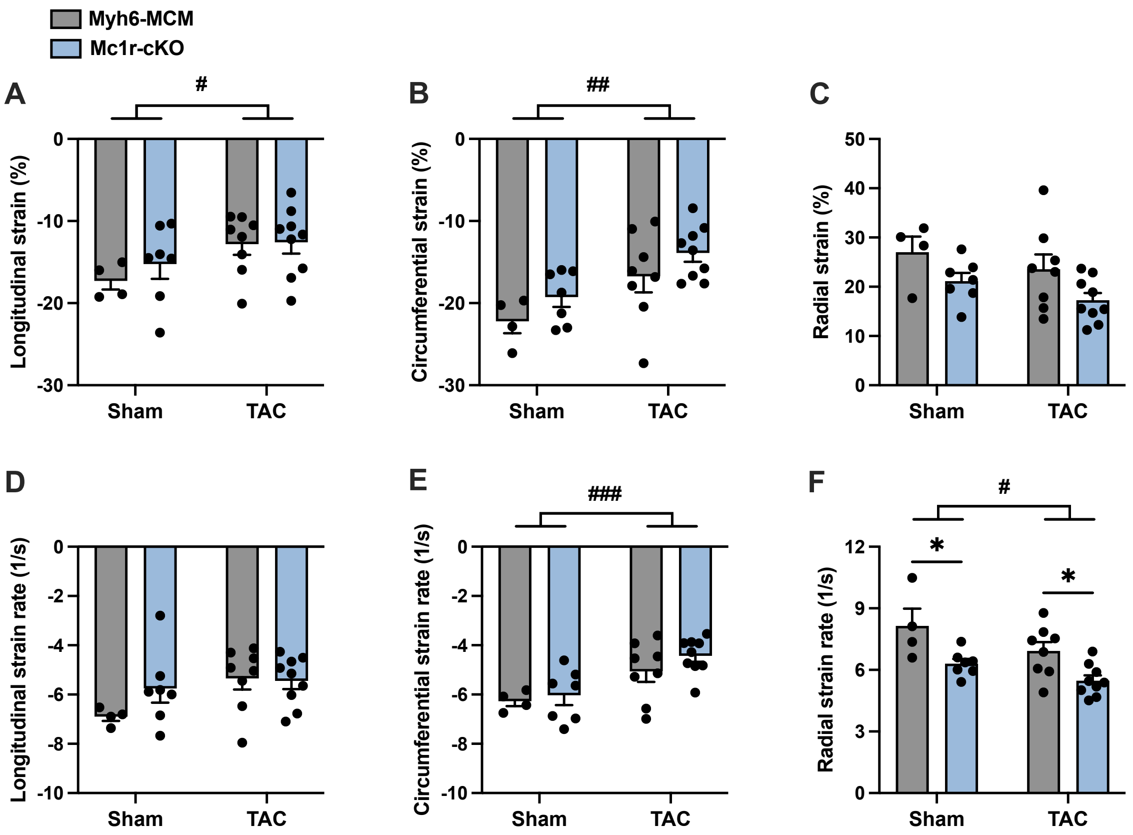

Figure S6. Strain and strain rate in sham- and TAC-operated Mc1r-cKO mice. (**A-C**) Speckle tracking-based analysis of global longitudinal strain (parasternal long axis) and global circumferential and radial strain (parasternal short axis) in Myh6-MCM and Mc1r-cKO mice after 8 weeks of sham or TAC surgery. (**D-F**) Speckle tracking-based analysis of global longitudinal strain rate (parasternal long axis) and global circumferential and radial strain rate (parasternal short axis) in Myh6-MCM and Mc1r-cKO mice after 8 weeks of sham or TAC surgery. n=4-9 mice per group in each graph. * *P*<0.05 for the indicated comparisons by 2-way ANOVA and Šídák's *post hoc* tests. # *P*<0.01 ## *P*<0.01 and ### *P*<0.001 for the main effect of TAC by 2-way ANOVA. Data are mean ± SEM, each dot represents individual mouse.

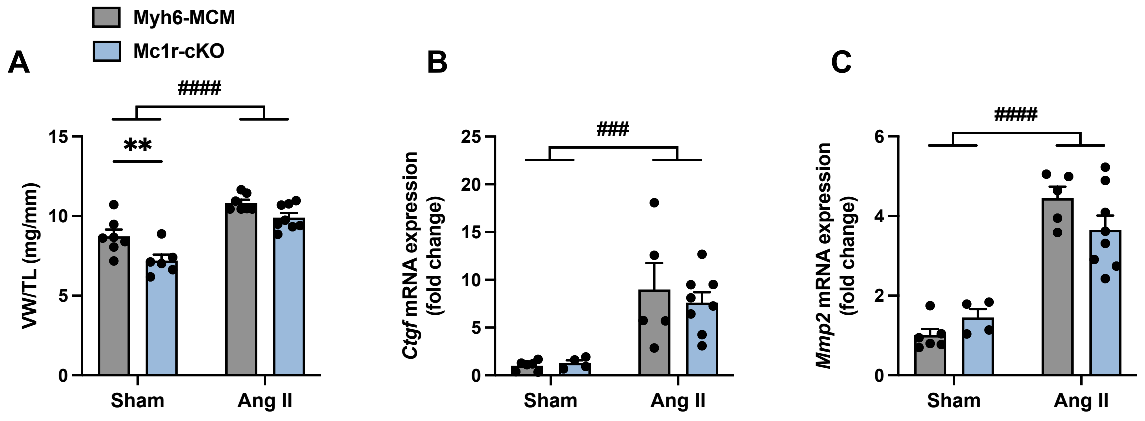

Figure S7. Relative ventricular weight and cardiac gene expression in Mc1r-cKO subjected to 4-week Ang II infusion. (**A**) Ventricular weight to tibia length ratio (VW/TL) in Myh6-MCM and Mc1r-cKO mice subjected to sham operation or Ang II infusion for 4 weeks. (**B**, **C**) qPCR analysis of *Ctgf* (connective tissue growth factor) and *Mmp2* (matrix metalloproteinase-2) in the LV of Myh6-MCM and Mc1r-cKO mice after 4 weeks of Ang II infusion. Gene expression is normalized against the geometric mean of *Actb* and *Mrps18a*. n=4-8 mice per group in each graph. ** *P*<0.01 for the indicated comparison by 2-way ANOVA and Šídák's *post hoc* tests. ### *P*<0.001 and #### *P*<0.0001 for the main effect of Ang II by 2-way ANOVA. Data are mean ± SEM, each dot represents individual mouse.

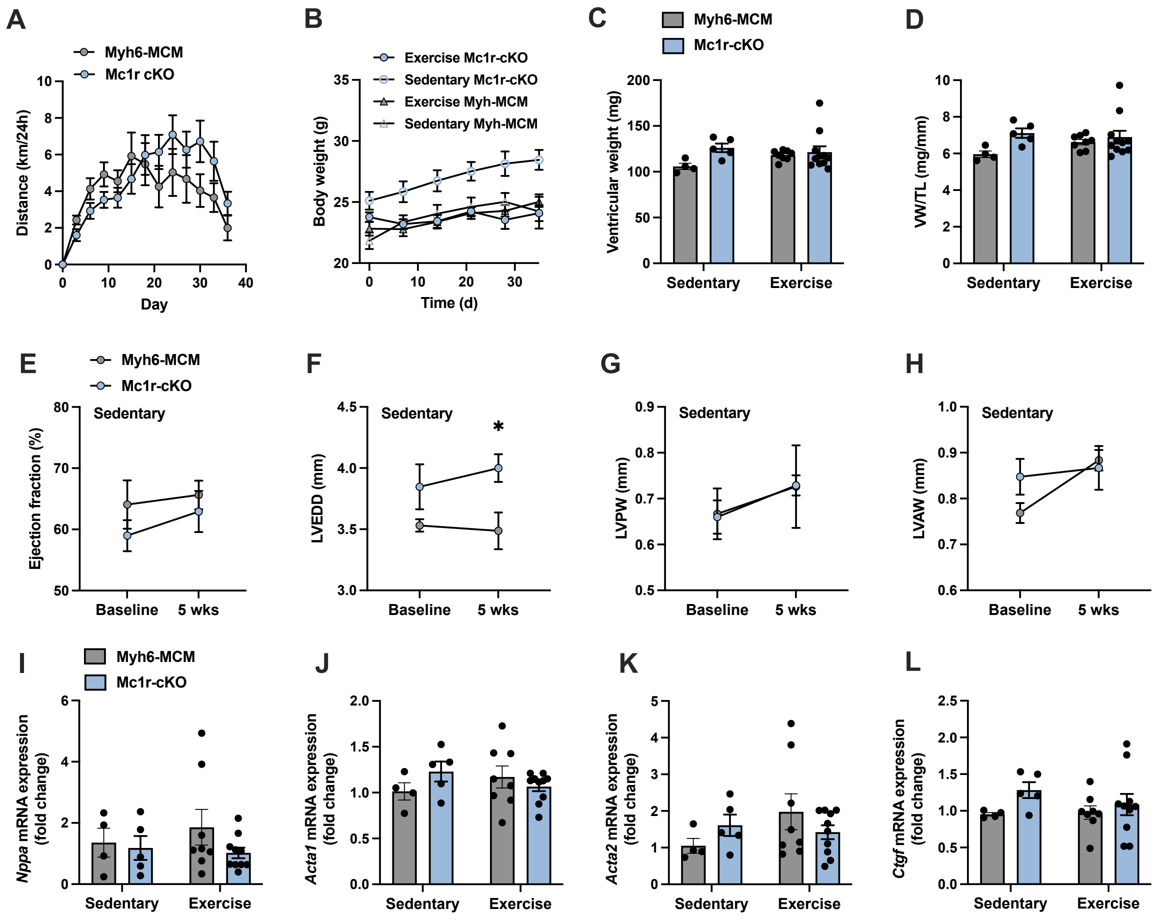

Figure S8. Ventricular weight, body weight, echocardiography and cardiac gene expression analysis in sedentary and exercising Myh6-MCM and Mc1r-cKO mice. (**A**) Average 24-hour running distance in Myh6-MCM and Mc1r-cKO mice subjected to voluntary wheel running for 5 weeks or a control non-exercising (sedentary) period. *n*=4-5 mice per group in the sedentary group, n=8 or 11 mice per group in the exercising group. The running distance data was analyzed using two-way repeated measures ANOVA, *P*=0.59 for genotype effect. (**B**) Body weight development in sedentary and exercising Myh6-MCM and Mc1r-cKO during the 5-week follow-up period. (**C, D**) Ventricular weight and ventricular weight to tibia length ratio (VW/TL) in Myh6-MCM and Mc1r-cKO mice at the end of the running wheel experiment. (**E-H**) Echocardiographic analysis of LV ejection fraction, LVEDD, LVPW and LV anterior wall thickness (LVAW) in sedentary Myh6-MCM and Mc1r-cKO mice at baseline and after the 5-week follow-up period. n=4-5 mice per group. * *P*<0.05 versus Myh6-MCM mice at 5 weeks by 2-way repeated measures ANOVA and Šídák's *post hoc* test. (**I-L**) qPCR analysis of *Nppa*, *Acta1*, *Acta2* and *Mmp2* in the LV of sedentary and exercising Myh6-MCM and Mc1r-cKO mice. Gene expression is normalized against the geometric mean of *Actb* and *Mrps18a*.

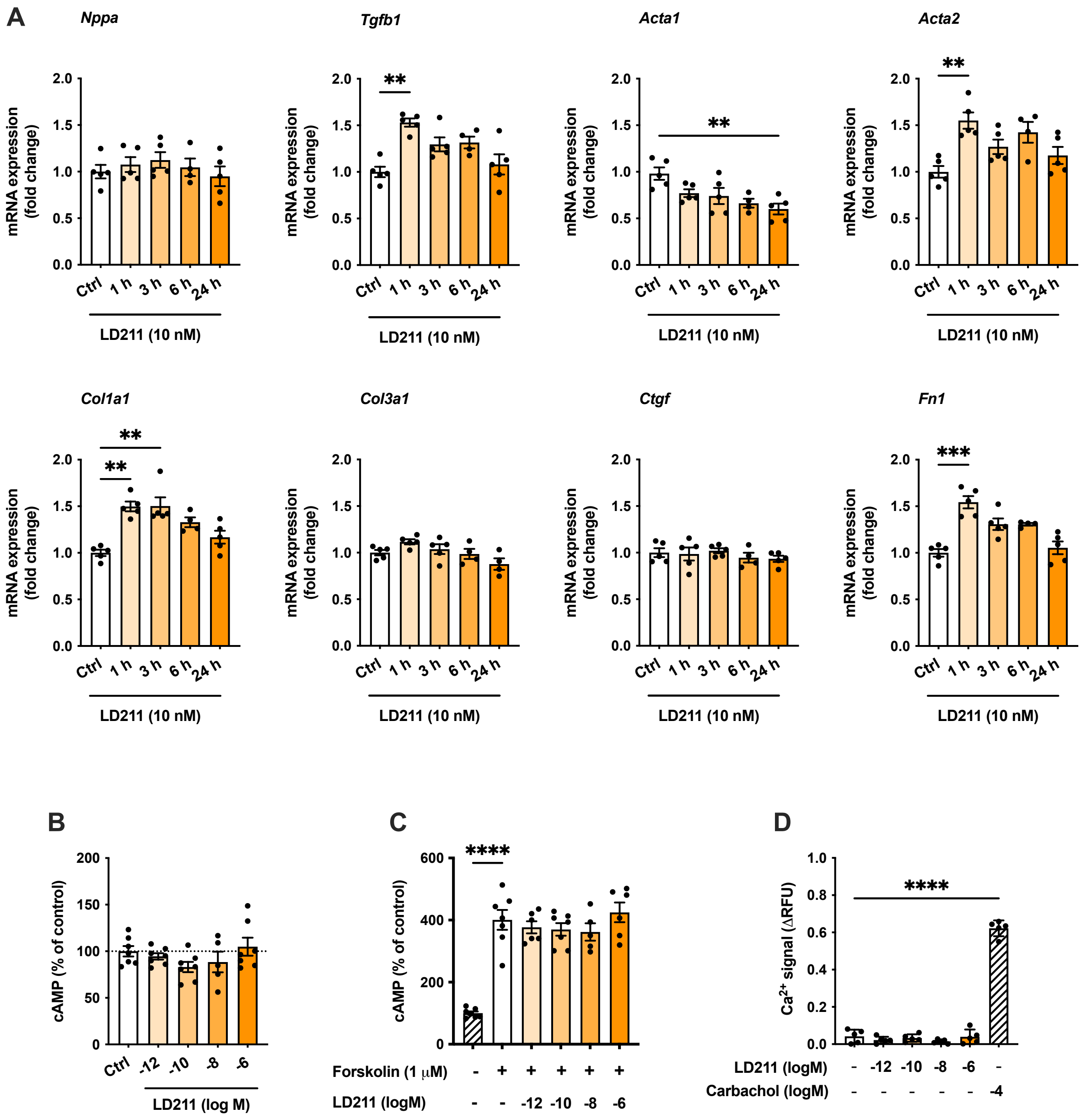

Figure S9. Selective activation of MC1R induces the expression of fibrosis-associated genes in neonatal mouse ventricular cardiac myocytes (NMCM). (**A**) qPCR analysis of *Nppa*, *Tgfb1*, *Acta1*, *Acta2*, *Col1a1*, *Col3a1*, *Ctgf* and *Fn1* in NMCMs treated with the selective MC1R agonist LD211 (10 nM) for 1, 3, 6 or 24 hours. Gene expression is normalized against the geometric mean of *Actb* and *Mrps18a*. ** P<0.01 and *** P<0.001 for the indicated comparisons by Kruskal Wallis and Dunn's *post hoc* tests. (**B, C**) Quantification of intracellular cAMP levels in H9c2 cells treated with different concentrations of LD211 for 30 minutes in the absence (**B**) or presence (**C**) of forskolin (1 µM). (**D**) Intracellular calcium signal in response to different concentrations of LD211 or carbachol (positive control) in H9c2 cells. **** P<0.0001 for the indicated comparisons by 1-way ANOVA and Dunnett *post hoc* tests. Data are mean ± SEM, n=4-7 per group in each graph.

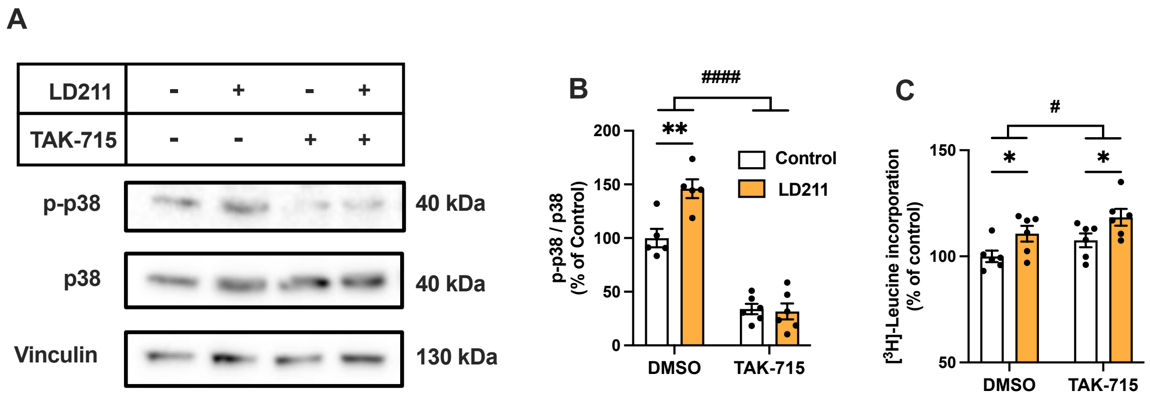

Figure S10. Inhibition of the p38 pathway does not block the hypertrophic effect of MC1R activation in cultured cardiomyocytes. (**A, B**) Representative Western blots and quantification of phosphorylated p38 (normalized to total p38) protein expression in H9c2 cells with or without the p38 inhibitor TAK-715 (5 µM) for 30 minutes followed by LD211 treatment (10 nM) for 15 minutes. (**C**) [^{3}H]-Leucine incorporation in H9c2 cells with or without the p38 inhibitor TAK-715 (5 µM) for 30 minutes followed by LD211 treatment (10 nM) for 24 hours. Data are mean ± SEM, n=*5*-6 per group in each graph. * *P*<0.05 and ** *P*<0.01 for the indicated comparisons by 2-way ANOVA and Šídák's *post hoc* tests. # *P*<0.05 and #### *P*<0.0001 for the main effect of TAK-715 by 2-way ANOVA.
